# A Versatile Panel of Reference Gene Assays for the Measurement of Chicken mRNA by Quantitative PCR

**DOI:** 10.1371/journal.pone.0160173

**Published:** 2016-08-18

**Authors:** Karen Staines, Ambalika Batra, William Mwangi, Helena J. Maier, Steven Van Borm, John R. Young, Mark Fife, Colin Butter

**Affiliations:** 1 The Pirbright Institute, Pirbright, Surrey, United Kingdom; 2 CODA-CERVA Veterinary and Agrochemical Research Centre, Brussels, Belgium; Universitat de Lleida, SPAIN

## Abstract

Quantitative real-time PCR assays are widely used for the quantification of mRNA within avian experimental samples. Multiple stably-expressed reference genes, selected for the lowest variation in representative samples, can be used to control random technical variation. Reference gene assays must be reliable, have high amplification specificity and efficiency, and not produce signals from contaminating DNA. Whilst recent research papers identify specific genes that are stable in particular tissues and experimental treatments, here we describe a panel of ten avian gene primer and probe sets that can be used to identify suitable reference genes in many experimental contexts. The panel was tested with TaqMan and SYBR Green systems in two experimental scenarios: a tissue collection and virus infection of cultured fibroblasts. GeNorm and NormFinder algorithms were able to select appropriate reference gene sets in each case. We show the effects of using the selected genes on the detection of statistically significant differences in expression. The results are compared with those obtained using 28s ribosomal RNA, the present most widely accepted reference gene in chicken work, identifying circumstances where its use might provide misleading results. Methods for eliminating DNA contamination of RNA reduced, but did not completely remove, detectable DNA. We therefore attached special importance to testing each qPCR assay for absence of signal using DNA template. The assays and analyses developed here provide a useful resource for selecting reference genes for investigations of avian biology.

## Introduction

Measurement of mRNA by quantitative RT-PCR (qRT-PCR) is applied in a wide variety of different experimental contexts. Valid interpretation of results depends crucially on the elimination of irrelevant variation, especially random and systematic variation in recovery of RNA. In many experimental designs, it is appropriate to convert raw results into quantities relative to the measured amounts of other transcripts in the samples, internal reference transcripts, whose variation under experimental conditions is expected to be minimal. This process is usually referred to as normalisation. Ample evidence has accumulated showing that commonly used reference transcripts are not universally effective, because the proportion of transcripts they represent may be modulated under the experimental conditions being investigated. In these cases, attempted normalisation leads to systematic bias in the measurement of other transcripts. Consequently, it is necessary to select and validate appropriate reference transcripts whenever they are applied in a new experimental context. However, in spite of prominent promotion of these requirements, the avian research field has been slow to adopt these recommendations. Some recent avian gene expression experiments have identified GAPDH, beta actin and 28s as stable reference genes during specific viral infections of particular tissues and cells [1–3], and others as suitable for immune cells subject to mitogen stimulation [4]. Although sets of candidate reference transcript assays are also offered commercially, their providers have chosen not to reveal full details of their design, contravening the basic requirement for independent verification. No set of reference genes is likely to be suitable for every different experimental condition and for this reason that we have developed a panel of candidate genes and reference assays, from which a subset of stable genes can be chosen for each experimental context.

A variety of algorithms are available for the selection of the most stable reference genes for a particular set of experimental samples. Two of these which have been widely used are the geNorm algorithm [5] and NormFinder [6]. Each starts with a panel of candidate genes, whose transcripts are expected to be present at constant levels. Measurements of these transcripts in samples representing the experimental variation are then compared using algorithms to estimate the stability of their levels across the samples. Whilst geNorm uses a single experiment-wide stability measure, NormFinder uses a method that balances variability within and between experimental groups. Both are dependent on assumptions about the candidate assays: geNorm assumes there is no correlation between levels of different candidates, treating inter- and intra- group variation separately. NormFinder deals with correlated expression by treating inter and intra-group variation separately, but still depends on the assumption that the average levels of all the genes are stable. NormFinder can also identify pairs of genes that may be more stable because of complementing inter-group variation.

Both these methods must start with a set of candidate transcripts for which these assumptions have a high likelihood of validity. The aim of this study was to contribute to the development of a reliable set of assays for chicken transcripts that would provide a suitable candidate set for use in many experimental applications.

## Materials and Methods

### Ethics Statement

All animal procedures were performed in accordance with the UK Animals (Scientific Procedures) Act 1986 [7]. This study was approved by the Pirbright Institute Ethical Review Panel and the UK Home Office.

### Experimental animals

ADOL (Avian Disease and Oncology Laboratory, East Lansing) Line O chickens and Rhode Island Red chickens (RIR) were obtained from the Pirbright Institute SPF breeding facility, from parents negative for antibodies to specified pathogens, and were kept in controlled-environment isolation rooms with food and water provided *ad libitum*. Birds were killed using Schedule 1 methods before harvesting tissues.

### Total RNA extraction from tissues

Samples of each of twelve tissues were collected from six 4 week old birds. These were selected to include those with primary lymphoid function (thymus, Bursa of Fabricius, spleen, caecal tonsil), muscular tissue (heart), dominated by epithelial cells (liver, kidney, lung), tissues that interface with the gut content (duodenum, ileum, colon) and the external environment (skin). Approximately 500 mg of tissue were cut into pieces of no more than 1mm across, collected into 2.5 ml of RNA Later (Ambion, UK), stored at 4°C overnight and then at -20°C for a maximum of 10 days prior to processing. RNA was extracted from 100 mg samples, using the Trizol Plus RNA Purification kit (Life Technologies), according to the manufacturer’s instructions. Homogenisation was performed using a Mixer Mill MM300 (Retsch) and 3 mm stainless steel cone balls (Retsch). An on-column DNAse digestion step was included (Purelink DNAse, Life Technologies). RNA purity was confirmed by measurement of the A_260_:A_280_ ratio with a NanoDrop 1000 (Thermo Scientific), with all samples giving readings between 1.9 and 2.0. Whilst he majority of were diluted to have A_260_ approximately 1.0, some samples with low RNA yields were used at up to ten-fold lower A_260_.

### Chick Embryo Fibroblasts infected with Highly Pathogenic Avian Influenza virus

The *in vitro* infection of avian cells, particularly chick embryo fibroblasts (CEF) has become a favoured technique for the study of cell-autonomous transcriptional responses, including to influenza, where viral replication itself can also be conveniently assayed by qRT-PCR. CEF were prepared from each of 4 Line 0 embryos at 11 days of embryonation, plated at 2.4x10^6^ per well in 6-well plates in 3 ml bicarbonate buffered 199 medium (Sigma) and allowed to adhere for 16 hours at 37°C in humidified 5% CO_2_ after which 1 ml of fresh medium was added, with or without 4000 units of Interferon α (IFNα) (gift from Peter Staeheli). After further incubation for 3 hours, medium was replaced with 2 ml fresh medium containing 2.2 x 10^7^ pfu highly pathogenic H5N1 avian influenza virus (A/Swan/Germany/R65/06) at an MOI of approximately 10, or control medium without virus, for 6 hours_._

### Macrophage Cell Line

The chicken macrophage cell line HD11 was used as a source of RNA for assaying the amplification efficiency and linear dynamic range of each assay.

### Total RNA extraction from cells

After washing cell monolayers with cold PBSa, RNA was extracted, from CEF or the macrophage cell line HD11 using the MirVana miRNA extraction kit (Ambion) according to manufacturer’s instructions. Concentrations of RNA were adjusted to 500 μg/ml, based on A260 measured by Nanodrop. The RNA integrity numbers (RIN) of all samples were over 9.0, as determined on a 2100 Bioanalyzer (Agilent Technologies).

### DNA extraction

A spleen from an adult RIR chicken was macerated in sterile PBS and passed through a 100 μM cell strainer (Fisher, UK). Cells were resuspended in Histopaque 1119 (Sigma, UK), overlaid with Histopaque 1083, Histopaque 1077 and finally PBSa. Following centrifugation at 800 x g for 45 min, cells at the 1119/1083 interface were collected and washed twice in sterile PBSa. DNA was prepared from 3x10^7^ cells resuspended in 1.5 ml TNE buffer (10 mM Tris, 1 mM EDTA, 100 mM NaCl, pH8.0), lysed by addition of 1% SDS, and digested with 0.1 mg/ml proteinase K at 60°C for 16 hours. The solution was extracted with phenol chloroform, then chloroform. DNA was precipitated from the aqueous layer with 2.5 volumes of ethanol, spooled out and washed twice with 75% ethanol before being dissolved in TNE buffer at approximately 100 μg/ml. After treatment with 2 mg/ml RNAse for 1 hour at 37°C and a second proteinase K digest, DNA was extracted, precipitated and re-dissolved as above. This genomic DNA was mechanically sheared and purity confirmed, with A260:A280 ratios between 1.7 and 2.0.

### Initial reference gene selection and primer design

We identified a set of genes, commonly used as reference genes in diverse species, for which chicken orthologues were known.

Primer and probe design was undertaken in a two-step process: Candidate primer pairs were identified using Genscript (https://www.genscript.com/ssl-bin/app/primer) as this gave the best control over some selection parameters in the initial identification of potential oligonucleotides. In this respect Primer Express (Applied Biosystems, Foster City, California, USA) was too stringent to offer candidates for all the genes.

However, Primer Express did allow use of a more detailed criteria for the final stages of design. The melting points calculated by each of the programs were different, and we used the Primer Express values in the final selection. These primer pairs were experimentally assessed, by end-point PCR and agarose gel electrophoresis, with both RNA and DNA templates. For each gene, pairs producing only a single product band of the expected size, from RNA but not from DNA, were selected for use in qPCR. For 28s ribosomal RNA assays employed in some analyses, we used a primer-probe set described elsewhere [8].

### RT-qPCR

#### RT-qPCR with TaqMan Probe

Probes incorporated 5-carboxyfluorescein (FAM) at the 5′end and N,N,N,N′ tetramethyl-6-carboxyrhodamine (TAMRA) at the 3′end. Assays were carried out using the SuperScript III Platinum One-Step qRT-PCR kit (Invitrogen). Amplification and detection of specific products was performed with the 7500 Fast Real Time System (TaqMan®; Applied Biosystems) with the following cycle profile: 50°C for 5 min, 95°C for 2 min and then 40 cycles of 95°C for 3 sec and 60°C for 30 sec. Generally, 20 ng of RNA was used for each assay. Standard curves were made starting with larger amounts, so that the experimental values fell within the range of the standard curves. Dilutions were carried out in 100 μg/ml carrier RNA (Qiagen) in buffer AVE (Qiagen).

#### RT-qPCR with SYBR Green

Reverse transcription of 1 μg of total RNA was performed with the SuperScript III Reverse Transcriptase kit (Life technologies) using the manufacturer’s protocol and stored at -20°C.

The RT-qPCR was performed using Blue Precision MasterMix with ROX premixed with SYBR Green (Primer Design Ltd) following the manufacturer’s protocol for geNorm selection of reference genes. Amplification and detection of specific products were carried out with the 7500 Fast Real Time System (TaqMan®; Applied Biosystems) with the following cycle profile: 95°C for 10 mins and then 40 cycles at 95°C for 15 sec and 60°C for 30 sec. On completion of the assay, a dissociation stage was carried out for melting curve analysis.

### Analysis of experimental data

All samples used to assay each gene, including standard curves, were carried out on a single plate, using a single threshold setting in the ABI 7500 software, eliminating the need for inter-run corrections due to different effective thresholds.

For standard curves, ten-fold dilution series were made so that the range of observed quantification cycle (*Ct)* values encompassed those obtained for all experimental samples. Linear regression of *Ct* as a function of logarithm to base 2 (log2) of the relative template concentration was carried out with the *lm* function in R, providing the slope and its standard error. Log2 were used throughout, so that a slope of -1 is expected for perfect two-fold amplification per cycle, easing intuitive assessment. Regressions were carried out using the R function *plot*.*stds*, included in S1 Text.

Before further processing, all *Ct* of experimental samples were adjusted by dividing by the slope of the standard regression curve for the appropriate gene. The resulting value is the log2 of the amount of transcript present at the start of the assay, relative to the threshold amount. This compensates for efficiency differences between transcripts, so that equal differences in this measure represent the same ratio between samples, whichever transcript is considered. The threshold amount, whose log2, is the intercept of the regression, is arbitrary and subject to large errors because it is far outside the range of the standard curve points. Thus the absolute measure has no useful meaning, and only differences, giving the ratios between two samples, are useful. For the majority of assays, the 95% confidence interval for the slopes spanned 1.0, so that the correction was not strictly required, and simple *ΔΔCq* methods would also have been appropriate. However, the adjustment was included for all assays for the sake of consistency with those assays which had slightly lower efficiencies.

All statistical calculations were carried out using data on the logarithmic scale, converting log differences to ratios only after all calculations were complete. There are several reasons for avoiding the use of intermediate values produced by exponentiation: it greatly simplifies all calculations, especially those dealing with experimental error and it maintains the data in the native logarithmic domain generated by the PCR assay system. Both experimental error and biological variation of the measurements are generally normally distributed on the logarithmic scale, so that assumptions underlying some parametric statistical methods are valid only by keeping data on this scale. For data with high variability, the lower bounds of confidence intervals calculated on linear scale data can be below zero, which is nonsensical.

#### Evaluation of candidate reference genes

Selection of stable reference genes in either of the two experimental contexts was carried out using the implementations of geNorm and Normfinder algorithms in the R package NormqPCR [9]. This adds a stepwise selection procedure to the original Normfinder algorithm. Wrapper functions used for these analyses are provided and described in S1 Text. geNorm analysis, of both SYBR Green and TaqMan qPCR data was also performed using the qbase+ software version 2.6 (Biogazelle).

#### Statistical modelling

The *gls* function of the *nlme* package in R was used for comparing levels of transcripts in experimental groups, because it allows the selection and evaluation of a variety of different error models. The results indicated the use of different variances only per gene, constant across factors, in both the tissue dataset and the infected fibroblast dataset. The selected models were analysed with the *glht* function of the *multcomp* package. The over-conservative Bonferroni correction of p values for multiple testing was used throughout. Details are provided in the R functions in S1 Text, and a flowchart describing the workflow for analysis is provided in S1 Fig. Considerations for underlying principles and treatment of error/uncertainty are summarised in S3 Text.

All Ct data for standard curves and for experimental samples are provided in S1 File, in a form suitable for input to R.

## Results

### Selection and testing of stable reference gene primer and probe sets

After tests with prospective primer sets, some of these were discarded as they were clearly highly variable (data not shown), leaving ten candidate reference transcripts. Details of these are provided in Table 1.

**Table 1:** Candidate reference genes.

Gene	Full name	ENA/Genbank	Refseq	ENSEMBL
**ACTB**	Beta actin	L08165	NM_205518	ENSGALT00000015673
**B2M**	Beta-2-microglobulin	M84767	NM_001001750	ENSGALT00000040255
**HMBS**	Hydroxymethylbilane synthase	BX932073	XM_417846	ENSGALT00000000380
**HPRT1**	Hypoxanthine phosphoribosyl transferase 1	AJ132697	NM_204848	ENSGALT00000009843
**PGK1**	Phosphoglycerate kinase 1	L37101	NM_204985	ENSGALT00000012893
**PLA2**	Phospholipase A2 group IV A	U10329	NM_205423	ENSGALT00000008121
**PPIA**	Peptidylprolyl isomerase A (Cyclophilin A)	GQ849480	NM_001166326	ENSGALT00000044106
**RPL13**	Ribosomal protein L13	D26318	NM_204999	ENSGALT00000009974
**RPLP0**	Ribosomal phosphoprotein P0	L28704	NM_204987	ENSGALT00000011731
**TBP**	TATA box binding protein	D83127	NM_205103	ENSGALT00000037720

Primers and probe sets for these genes were selected as described in the methodology.

**Fig 1:**
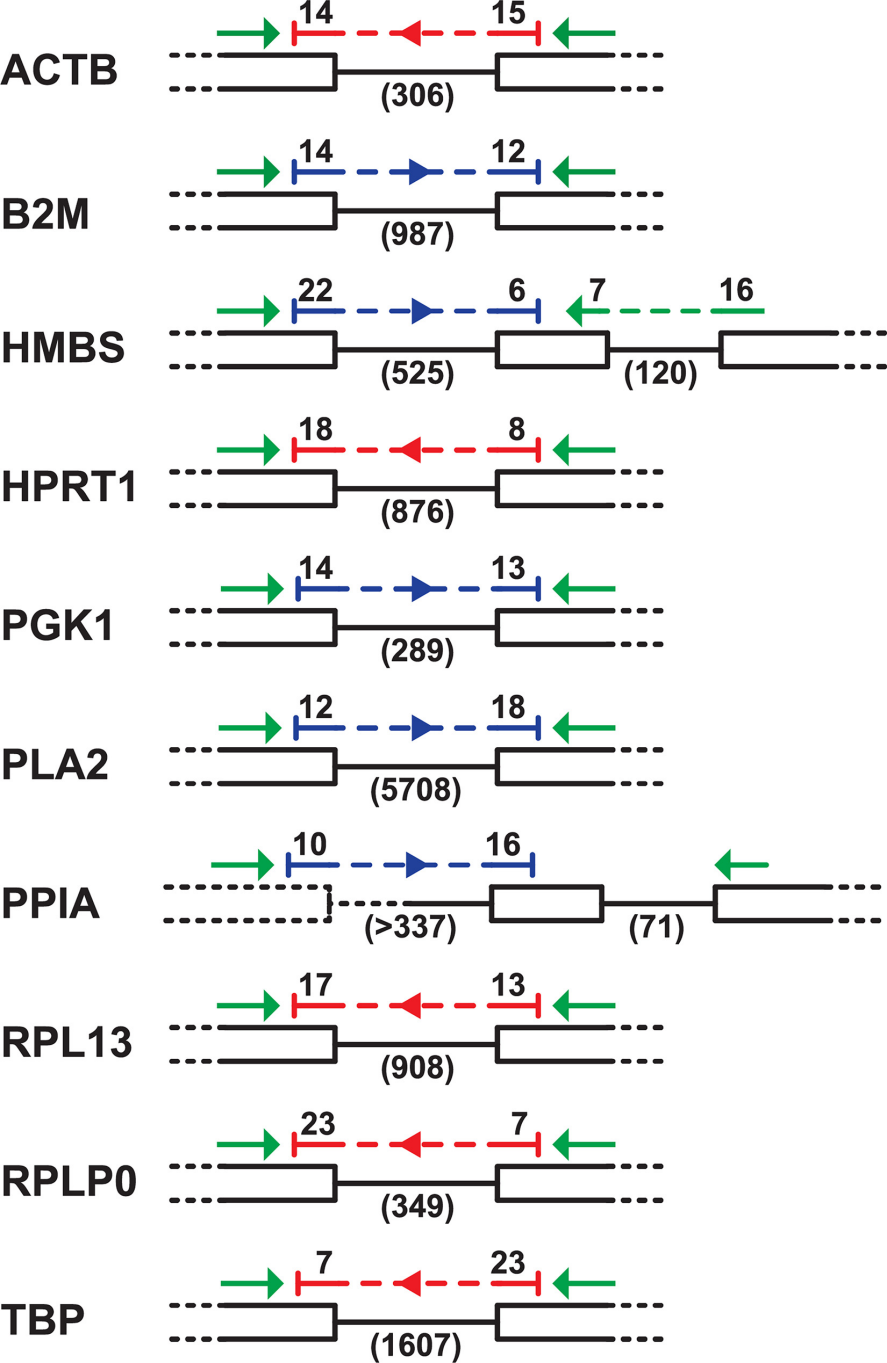
Layout of primers and probes for reference genes. Exons are indicated by black bars and introns by connecting lines. Dashed lines are sequences missing from the assembled genome sequence. Reading frames are all shown left-to-right. Primers are green arrows. Probes are blue or red for forward and reverse strand sequences respectively.

Results of tests with DNA templates showed that it was necessary to have the entirety of each primer within a different exon, so that the amplicon spanned an intron-exon boundary, excluding the amplification of DNA. Intron-spanning primers were not sufficient. Using these criteria, we also ensured that each probe sequence spanned the intervening intron, and we chose the larger of alternative introns when possible. The selected primers and corresponding probes are listed in Table 2. The layout of the selected oligonucleotides with respect to the spanned introns, and the intron lengths, are shown in Fig 1.

**Table 2:** Primer and probe sequences for qRT-PCR

Gene	Type	Sequence	Exon	Amplicon*
**ACTB**	Forward	5’-CAGGTCATCACCATTGGCAAT-3’	3	149/455
	Reverse	5’-GCATACAGATCCTTACGGATATCCA-3’	4	
	Probe	5’-(FAM)CACAGGACTCCATACCCAAGAAAGATGGC(TAMRA)-3’	3/4	
**B2M**	Forward	5’-AAGGAGCCGCAGGTCTAC-3’	2	151/1138
	Reverse	5’-CTTGCTCTTTGCCGTCATAC-3’	4	
	Probe	5’(FAM)CCGGGATGAGCACGGTCTGAAGAATT(TAMRA)-3’	3/4	
**HMBS**	Forward	5’-GGTTGAGATGCTCCGTGAGTTT-3’	3	153/798
	Reverse	5’-GGCTCTTCTCCCCAATCTTAGAA-3’	4	
	Probe	5’-(FAM)CCTGACCTCTGCTTTGAGATTGTTGCCA(TAMRA)-3’	3/4	
**HPRT1**	Forward	5’-TGGTCAAAAGAACTCCTCGAAGT-3’	6	96/972
	Reverse	5’-TGTAATCGAGGGCGTATCCAA-3’	7	
	Probe	5’-(FAM)TCCAACAAAGTCTGGCCGATATCCCA(TAMRA)-3’	6/7	
**PGK1**	Forward	5’-GTTTATGTCAATGATGCTTTTGGAA-3’	4	82/431
	Reverse	5’-GCCTTTGCAAAATAATCCAGTTCT-3’	5	
	Probe	5’-(FAM)CATCGTGCTCACAGCTCCATGGTAGGT(TAMRA)-3’	4/5	
**PLA2**	Forward	5’-GCACAAGACATTTGGCAGTTGT-3’	2	138/5846
	Reverse	5’-TGTGACATTTGTGGCTTTCCTTA-3’	3	
	Probe	5’-(FAM)CAACACATTGTGGTGGAACACCAGTACTCA(TAMRA)-3’	2/3	
**PPIA**	Forward	5’-CCCGTCGTGTTCTTCGACAT-3’	1	140/>450
	Reverse	5’-CCCTTGTAGCCAAATCCCTTCT-3’	2	
	Probe	5’-(FAM)CACCTTCGAGCTCTTCGCTGACAAGG(TAMRA)-3’	1/2	
**RPL13**	Forward	5’-TCGTGCTGGCAGAGGATTC-3’	3	71/908
	Reverse	5’-TCGTCCGAGCAAACCTTTTG-3’	4	
	Probe	5’-(FAM)TAATGCCCGCCAGTTTAAGCTCTTCTAGGC(TAMRA)-3’	3/4	
**RPLP0**	Forward	5’-TTGGGCATCACCACAAAGATT-3’	4	82/431
	Reverse	5’-CCCACTTTGTCTCCGGTCTTAA-3’	5	
	Probe	5’-(FAM)CATCACTCAGAATTTCAATGGTCCCTCGGG(TAMRA)-3’	4/5	
**TBP**	Forward	5’-CTTCGTGCCCGAAATGCT-3’	4	82/1689
	Reverse	5’-GCGCAGTAGTACGTGGTTCTCTT-3’	5	
	Probe	5’-(FAM)CTCATAATAACAGCAGCAAAACGCTTGGGA(TAMRA)-3’	4/5	

* Lengths of predicted amplicons from mRNA/DNA in base pairs

**Fig 2:**
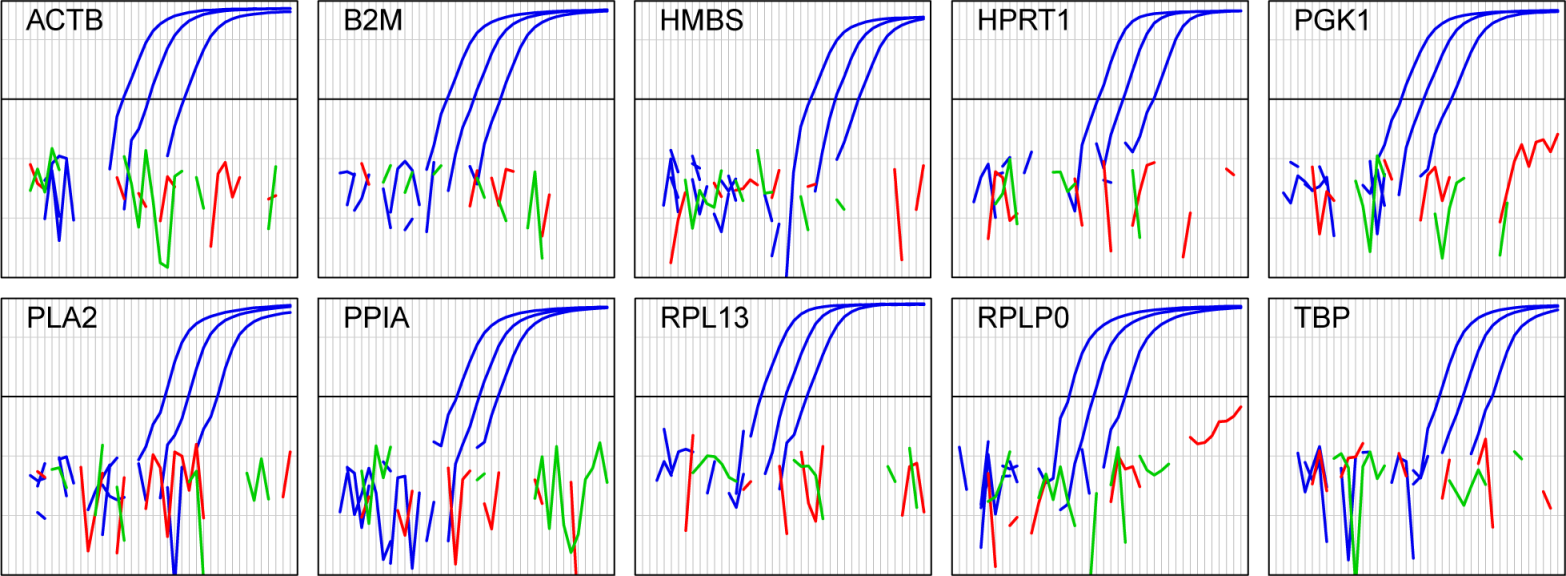
Primer-probe sets do not produce signals with DNA template. The primer-probe sets for the indicated transcripts were used in combined reverse-transcriptase-qPCR assays with HD11 cell line total RNA template (blue, 100, 10 and 1 ng), splenic DNA template (red, 20ng) or no template (green). The plots show background-subtracted fluorescence as a function of cycle (1–40) on a logarithmic scale. Missing points correspond to fluorescence levels lower than or equal to zero after automatic baseline subtraction. The heavy black line is the detection threshold used for all analyses.

The selected primer and probe sets were tested in the qPCR assays to ensure that they did not produce a signal with genomic DNA template (Fig 2). For all ten assays 10 ng of RNA gave a Ct value between 20 and 27, while 20 ng of DNA gave no signal even after the maximum 40 cycles. Thus even 100% DNA contamination of 20 ng RNA samples, such as those analysed in the experiments described here, would yield no signal from DNA.

**Fig 3:**
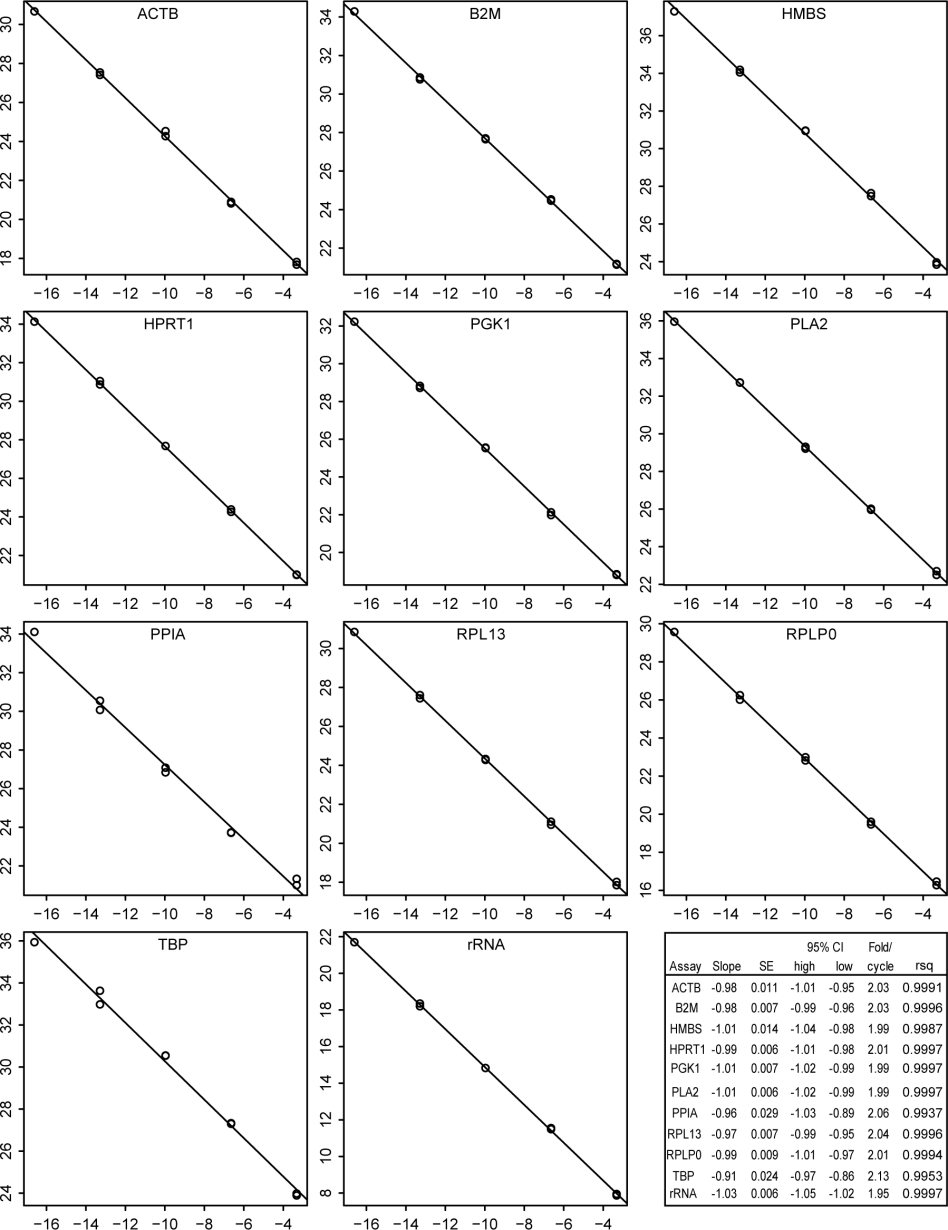
Linear regressions for efficiency of qPCR assays. Serial ten-fold dilutions of HD11 cell line RNA were analysed in duplicate for each candidate reference transcript. The Ct (y axis) were plotted against the log2 of the relative RNA concentrations (x axis). Names of transcripts are at the top of each graph. The inset shows the results of linear regression. SE is the standard error of the slope; CI indicates the 95% confidence interval; fold/cycle is the efficiency and RSq is the R squared value.

Amplification efficiency and linear dynamic range of each assay was evaluated by plotting standard curves of a dilution series of HD11 cell line total RNA, with the results shown in Fig 3. All regressions were linear over the range of the dilutions used, which included the transcript concentrations found in experimental samples. Slopes were very close to the -1.0 expected for perfect two-fold amplification per cycle. The accuracy of linearity over the full dilution range was dependent on the inclusion of carrier RNA in the dilution medium. Without carrier, the amplifications appeared to become less efficient at higher dilutions, possibly because of loss of RNA by adsorption on the walls of containing vessels. This effect was especially evident with ribosomal RNA assays, for which 100-fold higher dilutions were required because of the high abundance of the target. Essentially similar linearity and efficiencies were obtained using purified amplicon as template.

**Fig 4:**
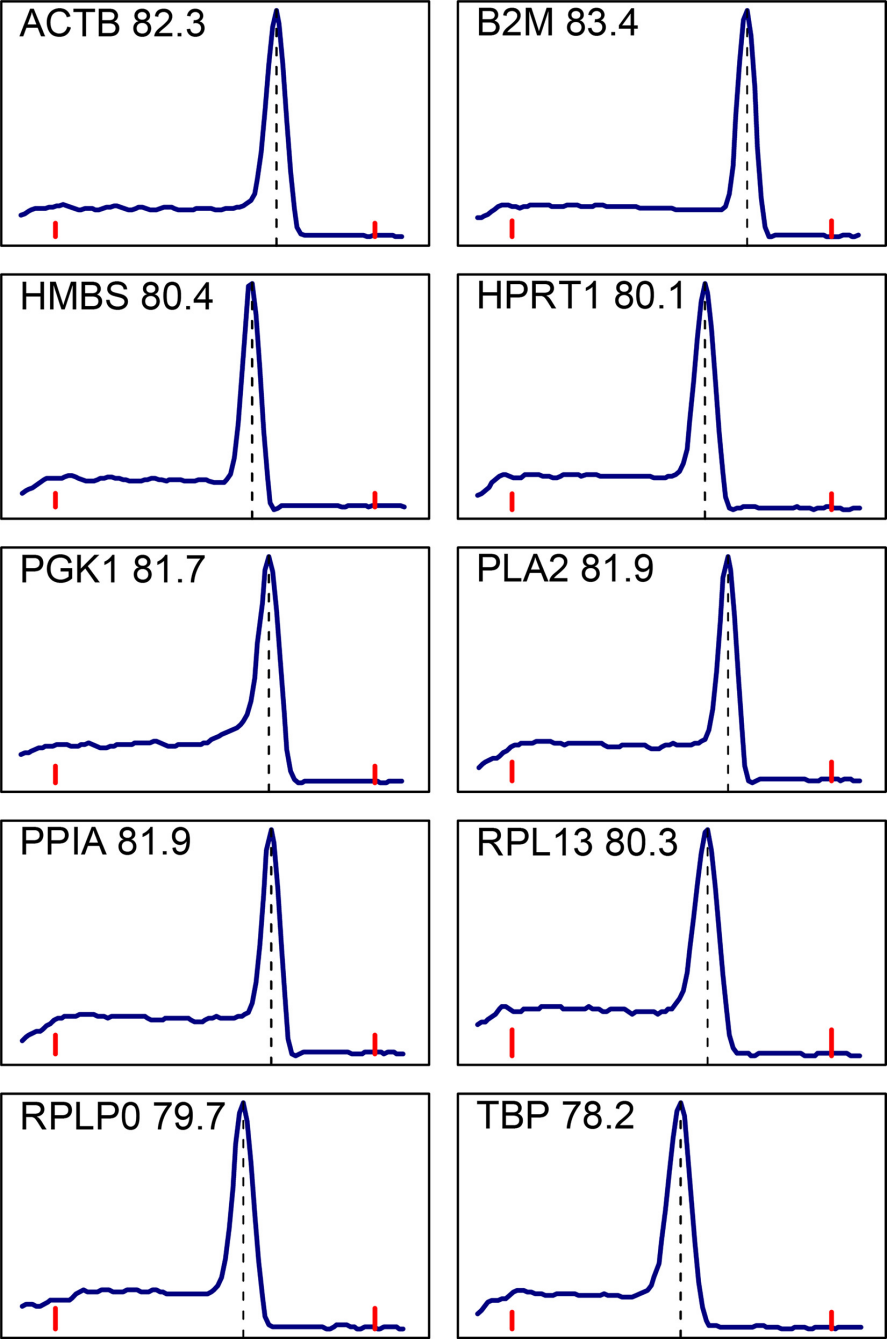
Melting curves of reference gene PCR products following SYBR Green assays. Blue curves show the rate of change of fluorescence as a function of temperature. Red bars indicate 65 and 90°C. The peak temperature for each curve is given next to the transcript name and is indicated with a vertical dashed line.

Dissociation curves of the amplified products of all SYBR Green assays were carried out to ensure their specificity. A typical result for each gene is shown in Fig 4. All exhibited a single sharp peak, indicating that only one product was amplified and that no primer dimers were formed.

### Identifying stably expressed reference genes

After verification of the quality of the assays, they were evaluated for their suitability as a set of potential reference transcript assays in two experimental scenarios, a panel of tissue RNA samples and samples of RNA from fibroblasts, with and without preincubation with interferon α before influenza virus or mock infection.

**Fig 5:**
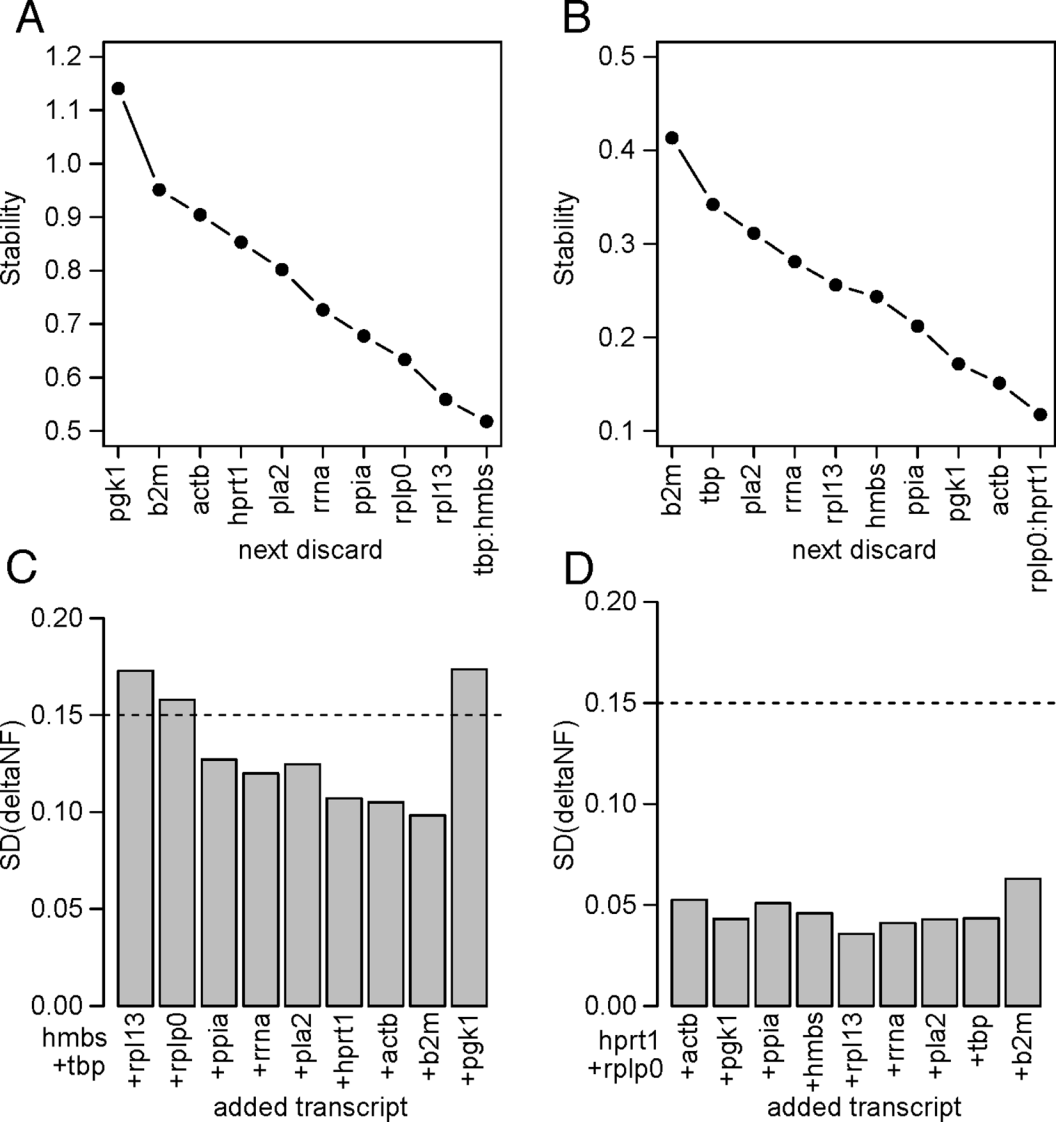
geNorm stepwise analysis of TaqMan analyses. Left (A, C), tissue panel; right (B,D), fibroblast/flu experiment. The upper graphs (A,B) show the change in stability measure [5] after eliminating the least stable gene, which is indicated on the x axis, at each step. The lower graphs (C,D) show the standard deviations of the differences in sample normalisation following successive additions of reference genes to the pool. Lowering of the bar is a measure of the improvement obtained by adding the indicated gene to the pool. The dashed line shows the 0.15 cut off level suggested as a criterion by Vandersompele [5] to stop adding genes.

The stability of the reference gene transcripts was analysed using the geNorm stepwise algorithm. For each of the two sample sets, raw Ct values from TaqMan assays for each gene on each sample were analysed using the stepwise geNorm algorithm. Fig 5 shows the results of successive elimination of the least stable transcripts in each experiment. For the tissue samples, the graphs (A and C) were similar to those described by Vandesompele [5]. Four genes (HMBS, TBP, RPL13 and RPLP0) were included before the next addition took the bar below the threshold criterion of 0.15 on the lower graph. There was an exceptional increase upon addition of the last gene, PGK1, indicating substantial instability because of differential expression of this transcript between the samples or tissues. This is consistent with the sharp drop in the stability parameter upon elimination of this gene evident in Fig 5. As subsequent analysis confirmed substantial differences in PGK1 transcript levels, especially in muscle, both geNorm and NormFinder were re-applied omitting this gene before normalisation for downstream analysis. The 28s rRNA assay was also omitted, as its use would require additional dilutions, and thus additional opportunity for error. Neither omission changed the order of the first five genes selected by geNorm.

With the fibroblast data, the geNorm cut-off criterion is already reached with just two genes in the pool, HPRT1 and RPLP0. The stability values were all better than the best obtained for the tissue panel data, possibly reflecting the homogeneous nature of the cell line compared with the complexity of the tissue samples.

While removal of the clearly differentially expressed PGK1 from the input for geNorm for the tissue data made no difference to the ranking of the remaining genes, this was not the case with NormFinder. With PGK1, the NormFinder ranking was RPL13, RPLP0, TBP and HMBS, the same four top ranked genes as with geNorm, but in different order. When PGK1 was excluded, NormFinder selected TBP, RPL13, HMBS and ACTB. Similarly with the infected fibroblast data, removal of B2M, the least stably expressed candidate reference gene in this experiment, made no difference to the geNorm ranking, but changed the ranking by NormFinder from HPRT1, HMBS, RPLP0, TBP, with B2M, to HPRT1, RPLP0, ACTB, PGK1, without B2M. The latter was the same as the geNorm rankings.

#### Normalisation of fibroblast interferon and infection data

**Fig 6:**
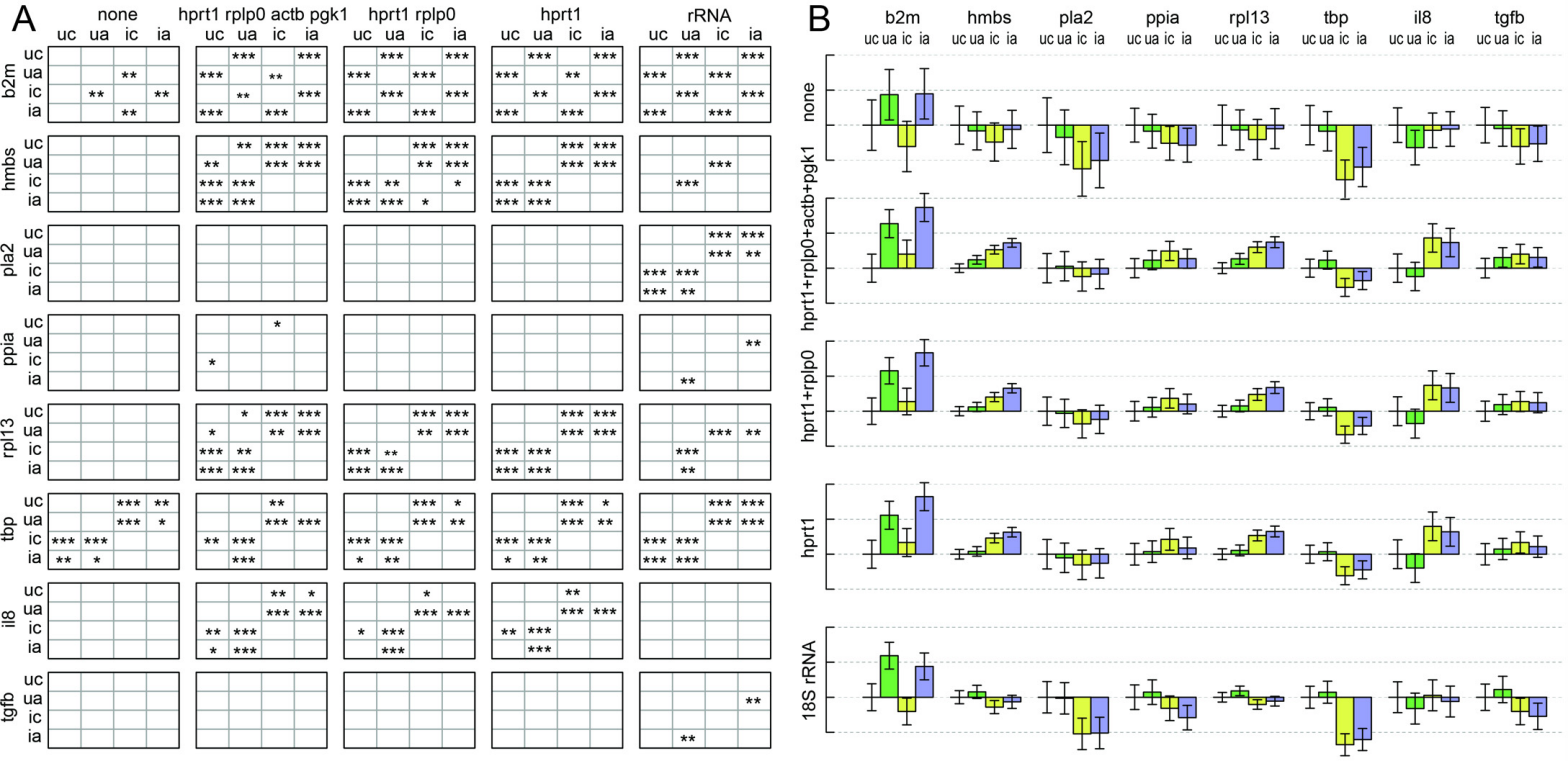
Effect of reference genes on detection of differential expression in an influenza-infected fibroblast experiment. **(A)** The title above each column indicates the set of genes used as reference genes for normalisation. Each row shows the results of statistical analysis for the gene indicated to the left. Experimental groups labelled 'u' or 'i', for uninfected and infected, and 'a' or 'c' for preincubation with or without IFNα. Asterisks indicate p values for rejection of the null hypothesis that a gene is not differentially expressed in the groups indicated by row and column labels; *** = p<0.001, ** = p<0.01, * = p<0.05. **(B)** Estimated transcript levels relative to the uninfected control without IFNα. Dotted lines are at unit log2 intervals, representing two-fold changes. Bars represent means of biological replicates, with 95% confidence intervals indicated by error bars. Annotations show the transcripts used for normalisation (left margin), the transcripts assayed (above) and the treatment, encoded as above.

The four top ranked genes, from either selection method after exclusion of the differentially expressed B2M, were used to normalise the measurements of the remaining six candidate transcripts, as well as IL-8 and TGFB transcripts, in the fibroblast infection dataset. The other candidate genes represent examples of genes with little difference in expression, providing a sensitive test of the effects of the normalisation on statistical analysis. Statistical modelling with gene-specific variance followed by conservative testing for significant differences in transcript levels (Bonferroni correction) was used to compare the results of normalisation with the selected genes or with 28s ribosomal rRNA. The results are shown in Fig 6, where asterisks indicate the strength of evidence for differential expression between experimental groups.

As expected from the diagnostic plots for the stepwise process in geNorm, in this experiment there is little difference between using the most stable gene, HPRT1, singly or in combination with further genes. The comparison is, however, useful in giving indications that a few of the differences may be less robust in the face of different normalisations than are others. When the results with reference gene normalisations are compared with those using rRNA as reference, the differences are striking. Although the differential expression of B2M remains clear, PLA2 appears to have no differential expression when compared with the selected reference genes, but clearly significant differences when compared with rRNA. Conversely, IL-8 appears differentially expressed when analysed using selected reference genes, but not when using rRNA. There are fewer differences with the other genes. So in this experimental context, comparison with the reference gene set and with rRNA will yield different conclusions about differential expression. It should be emphasised that the differences in expression here are small. For B2M, the maximum inter-group difference is estimated at about 3.5-fold or 3-fold with reference gene or rRNA normalisations, and the detection is unaffected by the choice. Estimates for PLA2 and IL-8, on the other hand, differ by up to about two-fold in the case where differential expression is detected and 1.2 to 1.3 fold where it is not.

All the estimated relative expression levels, and their confidence intervals, are shown in Fig 6B. It is clear that, compared with the reference normalisation, the relative level of rRNA in the infected cells is increased, so that using rRNA to normalise results in lower apparent levels of transcripts in these groups. That suppresses the increased expression of IL-8 evident with reference gene normalisation. It also causes the detection of significant decrease in the case of PLA2, which is not modulated at all with the reference gene normalised data. The effects of normalisation on individual samples, reduction of dispersion and removal of intra-group differences common to different genes, are illustrated in S2 Fig.

#### Normalisation of tissue panel data

**Fig 7:**
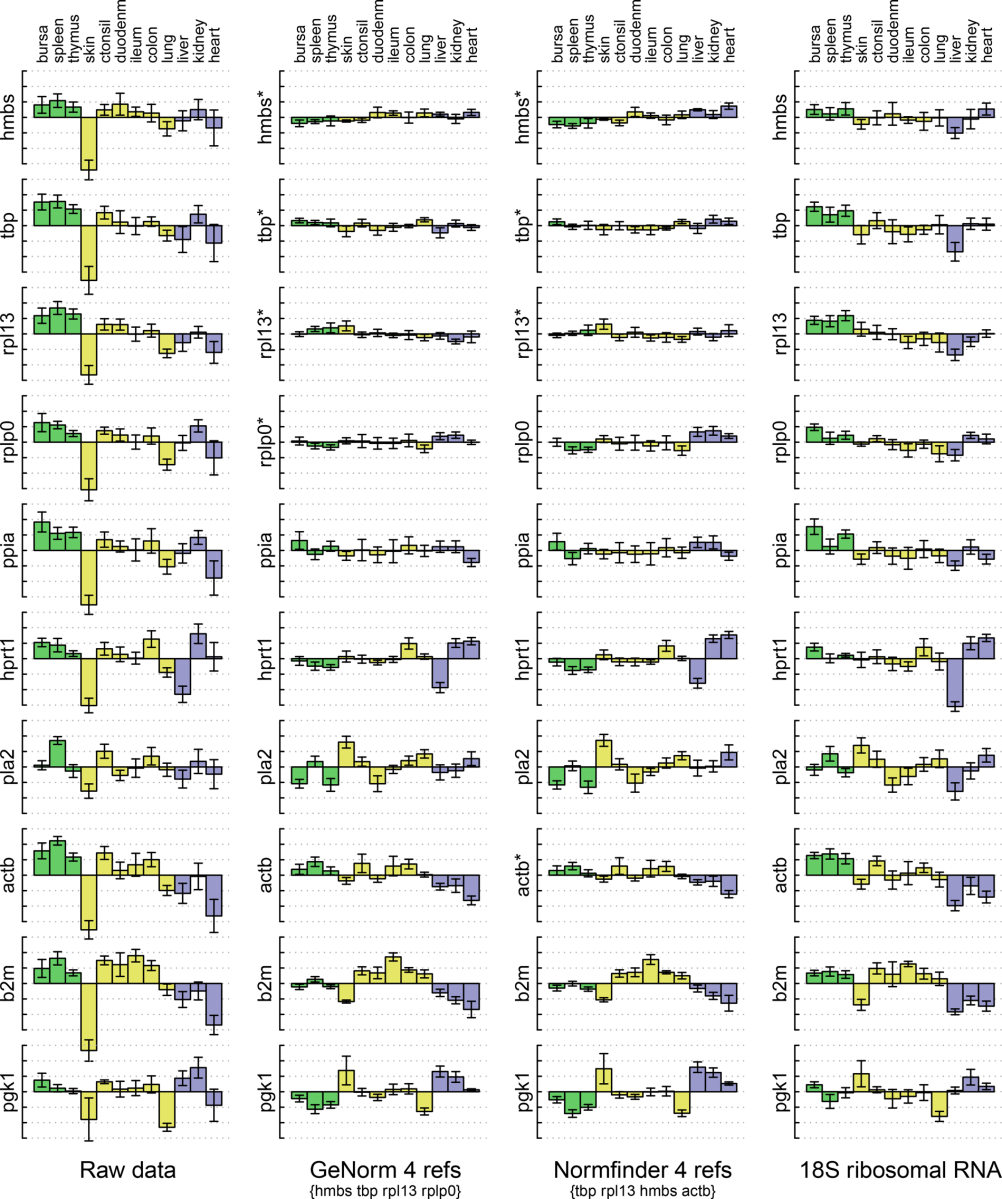
Transcript levels of candidate reference genes in tissues. Bars are means from samples from six birds, relative to the mean across tissues, plotted on a log2 scale. Dotted lines are at unit intervals, equivalent to 2-fold differences. Error bars are ± one standard deviation in each group, without pooling of variances. The left column shows un-normalised data. The remaining columns show the same data after normalisation with the reference genes indicated beneath, or with ribosomal RNA on the right. Measured genes are indicated to the left of each plot, marked with an asterisk where they were among those used for normalisation. Tissues are identified at the top. Colours of bars distinguish tissues with different categories of immunological function.

All the candidate reference transcripts and rRNA were measured in total RNA samples of thirteen tissues from six birds. The means and distributions of measurements, before and after different normalisations, are compared in Fig 7. Data from muscle samples is excluded here because the large differential expression of PGK1, responsible for its rejection as a reference candidate, would not fit on the scale used. All the normalisations substantially reduced the dispersion, represented by the error bars, of measurements from replicates of the same tissue, for all the measured genes, not just those used for normalisation. The means were also reduced in many cases, particularly the skin samples which were known, from A260 measurements, to have less RNA than other samples.

Reduction of dispersion of the data for the genes used for normalisation is an inevitable consequence of their use. However, the similar flattening out of the means for these genes shows that there is very limited inter-tissue variation, and thus that the selection of stable genes was successful. The PPIA data is similarly flattened although it was not used in the normalisation. Other genes show reduced but significant differences between tissues, including ACTB which was selected by NormFinder in place of RPLP0 selected by geNorm. Careful inspection of the ACTB profiles reveal that the inter-tissue differences are mostly in the opposite sense to those of the three genes selected by both algorithms, Thus the inclusion of ACTB provides an example of NormFinder's ability to select compensating deviations from stable expression.

Despite this difference, the results of normalisation are very similar for both reference gene selection algorithms using this dataset, as they were for the fibroblast data. Apart from the genes used in normalisation, normalisation with ribosomal RNA appeared to offer similar performance in reducing dispersion of levels among biological replicates. However, in spite of an overall similarity, for instance in removing the skin sample bias, there were some clear differences. Most obvious are the differences in the means for most genes in liver. These lead to differences in the detection of significant differential expression (S3 Fig) for genes like the unused candidate reference genes, where differences are small.

## Discussion

To be comparable across different experimental groups, measurements of transcript levels have to be expressed relative to a common base quantity. Although it is rarely explicitly identified, perhaps the most common implied basis is the level relative to the total number of mRNA transcripts.

It is in that context that the use of mRNA reference transcripts, whose low variability allow them to represent the total mRNA population, is appropriate. It is by now well established that one reference transcript is rarely sufficient, so that combinations must be used to attain low variation. Furthermore, it has been clearly demonstrated that there is no universal combination suitable for all investigations, and thus that a suitable combination has to be selected for every new experimental context. This means that a versatile panel of potentially appropriate candidate assays is required. We generated a suitable panel of ten assays with high amplification efficiencies and specificity, free from interference from contaminating genomic DNA, for use in chickens. In the course of that development we found that it was essential to verify the lack of genomic DNA amplification experimentally rather than relying on primer design rules. We also found that standard curves showing the best linearity over a large dynamic range required the presence of carrier RNA in the diluent. With these precautions we were able to show that the panel allowed the selection of suitable reference genes using established methods, and that their use yielded improvements in minimising variation, and greater sensitivity in detection of differential expression, in two experimental contexts. As expected, different combinations of transcripts were appropriate for each context because different members of the panel were excluded because of differential expression in the two systems.

It is important to emphasise that the reference genes selection results reported here cannot be extrapolated without explicit testing in every case. For example, it should not be assumed that reference genes suitable for this tissue panel will be suitable for a different selection of tissues, or for birds of different genetics or age. There is no alternative to testing in each new experimental context. Similarly, the reference genes that work best in the fibroblast infection experiment we analysed should not be assumed to be appropriate for other time points, other viruses, other interferon treatments, or even for fibroblasts from other lines of chickens.

The 28s ribosomal RNA assay we used here has been used for normalisation in a wide variety of investigations in chickens. For transcripts with very small differences in levels, exemplified here by the unselected candidate genes in each system, we found that there could be differences in the detection of differential expression using this instead of the selected reference mRNAs for normalisation. However, this does not imply that all analyses that used the 28s rRNA assay are suspect. First, the greatest differences we saw were less than four-fold, and most much smaller. So only those conclusions based on very small differences are open to reinterpretation. Second, in very many cases, the biological interpretation depends not on the ratios of one transcript in different groups, but rather on the ratio of different transcripts. An example of this in avian science is to be found in the developing bursa, where the requirements for B cell survival appear to change, dependent on the ratio of BAFFR and TACI mRNAs [10]. In those cases, the method used for normalisation does not affect the result, as the normalisation factor cancels out in the ratio of two other transcripts. Although our data are enough to prefer the use of mRNA reference genes in future, they do not imply that existing work using 28s RNA should be rejected.

In the case of the influenza infected fibroblasts, an increase of IL-8 mRNA in infected cells was evident using normalisation with the mRNA reference transcripts, but not using 28s rRNA. At first sight this appears to be a failure of the 28s normalisation, the rRNA having increased relative to the reference transcripts. However, it is known that infection with influenza virus causes a general suppression of host cell mRNA [11]. So the level of IL-8 mRNA per cell may indeed be unaltered, and if that is the quantity pertinent to biological interpretation, then it is the 28s rRNA that may provide the more appropriated normalisation. This case illustrates the danger of unthinking adherence to prescriptions for data analysis prescribed in manifestos such as the MIQE [12–15]. Generalised guidelines are of great value in drawing attention to the important considerations, but are not a panacea to be followed blindly. They cannot absolve author or referee from the responsibility for critical evaluation of analyses in each specific case.

There are many other experimental situations where employment of the normalisation strategies described here are inappropriate. For example, when assays are carried out in cell cultures in which cell populations may be rapidly changing by cell death and/or proliferation of bystander cells in response to experimental variables, internal reference normalisation may completely obscure effects confined to a stable subset of cells. In such cases, the appropriate basis may be the amount of transcript per culture well. If RNA recovery is significantly variable, spiking with cells carrying a unique reference transcript, immediately prior to RNA extraction, can provide a more appropriate normalisation. In infected tissues, whose cellular composition and size may vary dramatically with experimental conditions, the relevant basis may be numbers of mRNA molecules per gram of tissue, or even number in the whole tissue. Neither of these is likely to be well represented by any internal reference transcripts, so that spiked references may again be required if the ultimate scientific interpretation depends on the control of variation in RNA recovery.

The original NormFinder implementation [6] provided for selection of only up to two reference genes, although the publication described the mathematical basis for evaluating combinations of more than two. The NormqPCR package provided a stepwise procedure implementing those calculations. However, there are two ways the NormqPCR stepwise process might be improved. First, because of the possibility of compensation of inter-group differences between pairs of genes, the best combination of a number of genes does not necessarily include those in the smaller combinations, but the NormqPCR procedure always includes them. Second, by not eliminating the least stable gene an opportunity is missed to reduce bias in the overall average expression, and thus in the selection, by elimination of candidates with the greatest individual inter-group differences. We implemented a new version of the stepwise procedure that tested all combinations of increasing size, not just those including the previously selected smaller combination. That was then enclosed within another stepwise loop that successively eliminated the least stable gene from the initial set of candidates. Parameters were also added to set a minimum individual stability for inclusion in the combinations, either by stability value or by rank, as was provided for the pairwise combinations in the original NormFinder procedure. The output provides stability measures for all examined combinations, as well as the output that would be produced by the NormqPCR procedure, for each iteration of the outer loop. This procedure is provided in S2 Text, and example results in S4 Fig. In general the improvement in stability obtained with the new procedure, compared with the existing NormqPCR procedure, was small enough that effects on the statistical analysis of experimental data were not useful. Therefore the results presented here are those using the combinations selected by the simpler NormqPCR procedure.

Robustness of reference gene selection in the tissue data was tested by removing one tissue at a time from the panel. The geNorm selection was relatively robust, while ranking by NormFinder was much more sensitive to these changes in the input data (S6 Fig). It is likely that the advantage of NormFinder in being able to select reference genes with compensating inter-group differences for a given dataset may come at the cost of decreased robustness with different input data. This may be a factor in choosing between these selection algorithms depending on the intended application.

Threshold based standard curve methods have been criticised because of their assumption of constant PCR efficiency in the cycles preceding achievement of the threshold signal, and potential variability of efficiency between samples [16]. In response, several methods have been developed based instead on the estimation of efficiencies in individual amplification curves. These are compromised by their dependence on fitting data obtained after achievement of the detectable signal, inevitably approaching or including the non-exponential phase of amplification, so that they cannot assess the progress of the amplification in the preceding part of the reaction except by extrapolation. Lack of linearity of standard curves based on dilution series [17] and lower efficiency at higher dilution have been advanced as reasons to support the use of these alternatives. In our experiments we found that inclusion of carrier RNA in dilution medium dramatically improved the linearity of standard curves and improved some otherwise apparently low efficiencies, especially at the high dilutions used for the rRNA assay. Thus major questionable characteristics of standard curves, used to justify rejection of the assumption of constant amplification efficiency, may be attributable to adsorbtion of nucleic acids on the walls of dilution vessels. This would also be expected to result in higher variance at higher dilutions, an effect documented by Boggy & Woolf [17]. We are not aware of others having emphasized the improvements obtainable with carrier. Whether or not our hypothetical explanation for the improvement is correct, the standard curves we obtained demonstrated excellent linearity over ranges of starting template concentrations including all experimental values, justifying use of the constant efficiency assumption and thus of the applicability of the threshold-based method of quantification.

A general schematic for the design and application of reference gene assay is given in S5 Fig. In summary, we have described a rigorous process by which to design qPCR primer and probe sets for mRNA transcripts; shown the importance of experimentally determining their inability to amplify genomic DNA; provided a panel of reference gene assays from which to select by experiment those with best utility for particular applications; described their use in the normalisation of experimental data and lastly have discussed when approaches other than this may better fit the biology of particular experiments.

## Supporting Information

S1 Fig Data analysis workflow. (PDF)

S2 Fig Residual plots of flu infection experiment data. (PDF)

S3 Fig Effect of normalisation on detection of significant differences in tissue panel. (PDF)

S4 Fig Nested loop NormFinder reference gene selection. (PDF)

S5 Fig Reference gene assay design and evaluation flowchart. (PDF)

S6 Fig Robustness of candidate reference gene selection. (PDF)

S1 File Raw Ct data. (ZIP)

S1 Text Rfunctions used for data analysis. (TXT)

S2 Text R functions for nested loop NormFinder selection. (TXT)

S3 Text Data analysis supplement. (PDF)
