# Supplementary material for: A Versatile Panel of Reference Gene Assays for the Measurement of Chicken mRNA by Quantitative PCR
Source: PLoS One. 2016 Aug 18;11(8):e0160173. doi: 10.1371/journal.pone.0160173 (PMC4990416; doi:10.1371/journal.pone.0160173)
Supplement: S1 Fig — (PDF) [file pone.0160173.s001.pdf]

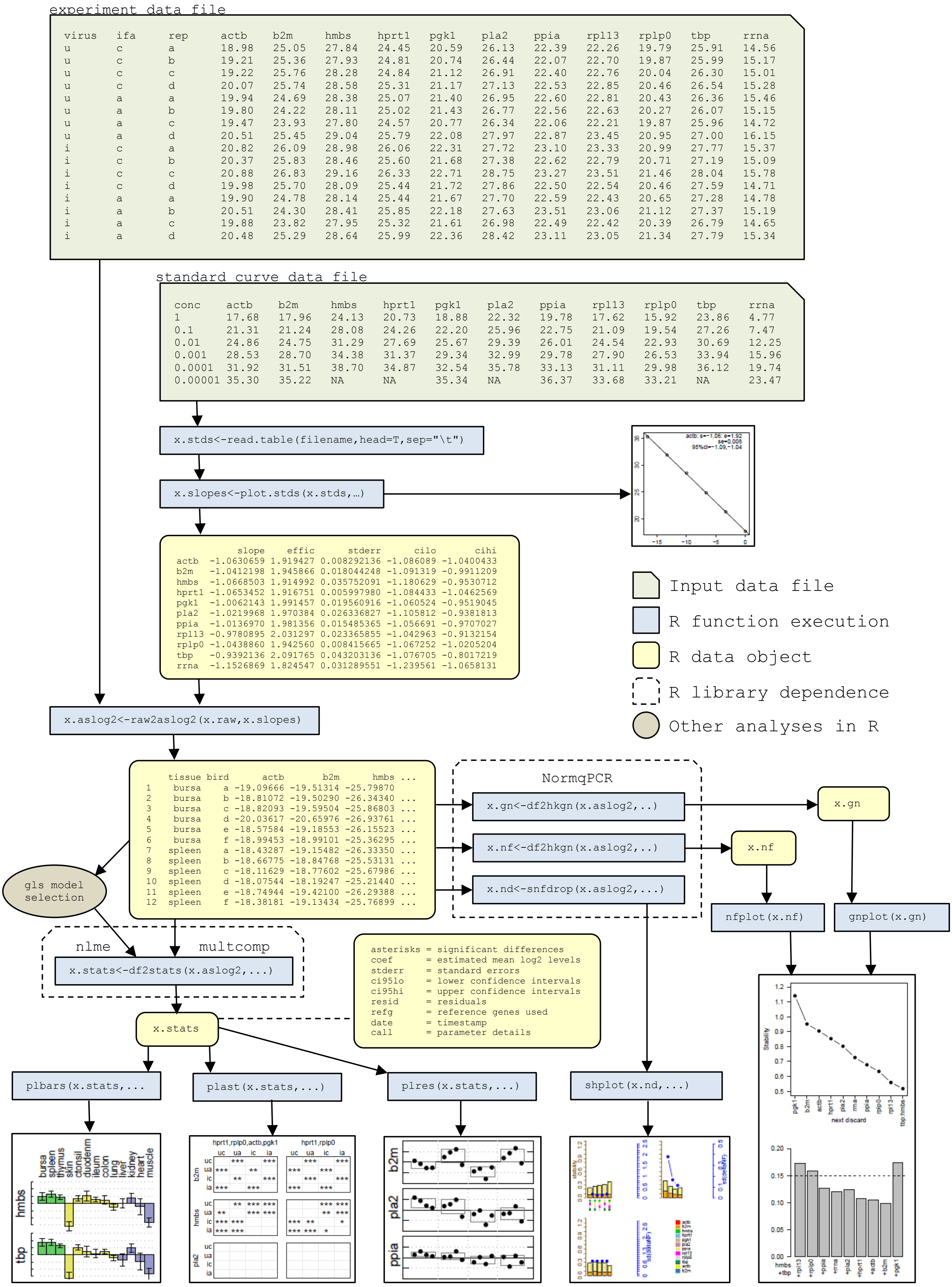

experiment data file
virus ifa rep actb b2m hmbs hprt1 pgk1 pla2 ppia rpl13 rplp0 tbp rrna
u c a 18.98 25.05 27.84 24.45 20.59 26.13 22.39 22.26 19.79 25.91 14.56
u c b 19.21 25.36 27.93 24.81 20.74 26.44 22.07 22.70 19.87 25.99 15.17
u c c 19.22 25.76 28.28 24.84 21.12 26.91 22.40 22.76 20.04 26.30 15.01
u c d 20.07 25.74 28.58 25.31 21.17 27.13 22.53 22.85 20.46 26.54 15.28
u a a 19.94 24.69 28.38 25.07 21.40 26.95 22.60 22.81 20.43 26.36 15.46
u a b 19.80 24.22 28.11 25.02 21.43 26.77 22.56 22.63 20.27 26.07 15.15
u a c 19.47 23.93 27.80 24.57 20.77 26.34 22.06 22.21 19.87 25.96 14.72
u a d 20.51 25.45 29.04 25.79 22.08 27.97 22.87 23.45 20.95 27.00 16.15
i c a 20.82 26.09 28.98 26.06 22.31 27.72 23.10 23.33 20.99 27.77 15.37
i c b 20.37 25.83 28.46 25.60 21.68 27.38 22.62 22.79 20.71 27.19 15.09
i c c 20.88 26.83 29.16 26.33 22.71 28.75 23.27 23.51 21.46 28.04 15.78
i c d 19.98 25.70 28.09 25.44 21.72 27.86 22.50 22.54 20.46 27.59 14.71
i a a 19.90 24.78 28.14 25.44 21.67 27.70 22.59 22.43 20.65 27.28 14.78
i a b 20.51 24.30 28.41 25.85 22.18 27.63 23.51 23.06 21.12 27.37 15.19
i a c 19.88 23.82 27.95 25.32 21.61 26.98 22.49 22.42 20.39 26.79 14.65
i a d 20.48 25.29 28.64 25.99 22.36 28.42 23.11 23.05 21.34 27.79 15.34
standard curve data file
conc actb b2m hmbs hprt1 pgk1 pla2 ppia rpl13 rplp0 tbp rrna
1 17.68 17.96 24.13 20.73 18.88 22.32 19.78 17.62 15.92 23.86 4.77
0.1 21.31 21.24 28.08 24.26 22.20 25.96 22.75 21.09 19.54 27.26 7.47
0.01 24.86 24.75 31.29 27.69 25.67 29.39 26.01 24.54 22.93 30.69 12.25
0.001 28.53 28.70 34.38 31.37 29.34 32.99 29.78 27.90 26.53 33.94 15.96
0.0001 31.92 31.51 38.70 34.87 32.54 35.78 33.13 31.11 29.98 36.12 19.74
0.00001 35.30 35.22 NA NA 35.34 NA 36.37 33.68 33.21 NA 23.47
x.stds<-read.table(filename,head=T,sep="\t")
x.slopes<-plot.stds(x.stds,…)
actb: s=-1.06; e=1.92
se=0.008
95%ci=-1.09,-1.04
slope effic stderr cilo cihi
actb -1.0630659 1.919427 0.008292136 -1.086089 -1.0400433
b2m -1.0412198 1.945866 0.018044248 -1.091319 -0.9911209
hmbs -1.0668503 1.914992 0.035752091 -1.180629 -0.9530712
hprt1 -1.0653452 1.916751 0.005997980 -1.084433 -1.0462569
pgk1 -1.0062143 1.991457 0.019560916 -1.060524 -0.9519045
pla2 -1.0219968 1.970384 0.026336827 -1.105812 -0.9381813
ppia -1.0136970 1.981356 0.015485365 -1.056691 -0.9707027
rpl13 -0.9780895 2.031297 0.023365855 -1.042963 -0.9132154
rplp0 -1.0438860 1.942560 0.008415665 -1.067252 -1.0205204
tbp -0.9392136 2.091765 0.043203136 -1.076705 -0.8017219
rrna -1.1526869 1.824547 0.031289551 -1.239561 -1.0658131
Input data file
R function execution
R data object
R library dependence
Other analyses in R
x.aslog2<-raw2aslog2(x.raw,x.slopes)
tissue bird actb b2m hmbs ...
1 bursa a -19.09666 -19.51314 -25.79870 ...
2 bursa b -18.81072 -19.50290 -26.34340 ...
3 bursa c -18.82093 -19.59504 -25.86803 ...
4 bursa d -20.03617 -20.65976 -26.93761 ...
5 bursa e -18.57584 -19.18553 -26.15523 ...
6 bursa f -18.99453 -18.99101 -25.36295 ...
7 spleen a -18.43287 -19.15482 -26.33350 ...
8 spleen b -18.66775 -18.84768 -25.53131 ...
9 spleen c -18.11629 -18.77602 -25.67986 ...
10 spleen d -18.07544 -18.19247 -25.21440 ...
11 spleen e -18.74944 -19.42100 -26.29388 ...
12 spleen f -18.38181 -19.13434 -25.76899 ...
NormqPCR
x.gn<-df2hkgn(x.aslog2,..)
x.nf<-df2hkgn(x.aslog2,..)
x.nd<-snfdrop(x.aslog2,...)
x.gn
x.nf
nfplot(x.nf)
gnplot(x.gn)
gls model selection
nlme
multcomp
x.stats<-df2stats(x.aslog2,...)
x.stats
asterisks = significant differences
coef = estimated mean log2 levels
stderr = standard errors
ci95lo = lower confidence intervals
ci95hi = upper confidence intervals
resid = residuals
refg = reference genes used
date = timestamp
call = parameter details
plbars(x.stats,...
plast(x.stats,...)
plres(x.stats,...)
shplot(x.nd,...)
bursa
spleen
thymus
skin
tonsil
duodenum
ileum
colon
lung
liver
kidney
heart
muscle
hmbs
tbp
hprt1,rplp0,actb,pgk1
hprt1,rplp0
uc ua ic ia
b2m
hmbs
pla2
ppia
Stability
next discard
