# Supplementary material for: A Versatile Panel of Reference Gene Assays for the Measurement of Chicken mRNA by Quantitative PCR
Source: PLoS One. 2016 Aug 18;11(8):e0160173. doi: 10.1371/journal.pone.0160173 (PMC4990416; doi:10.1371/journal.pone.0160173)
Supplement: S2 Fig — (PDF) [file pone.0160173.s002.pdf]

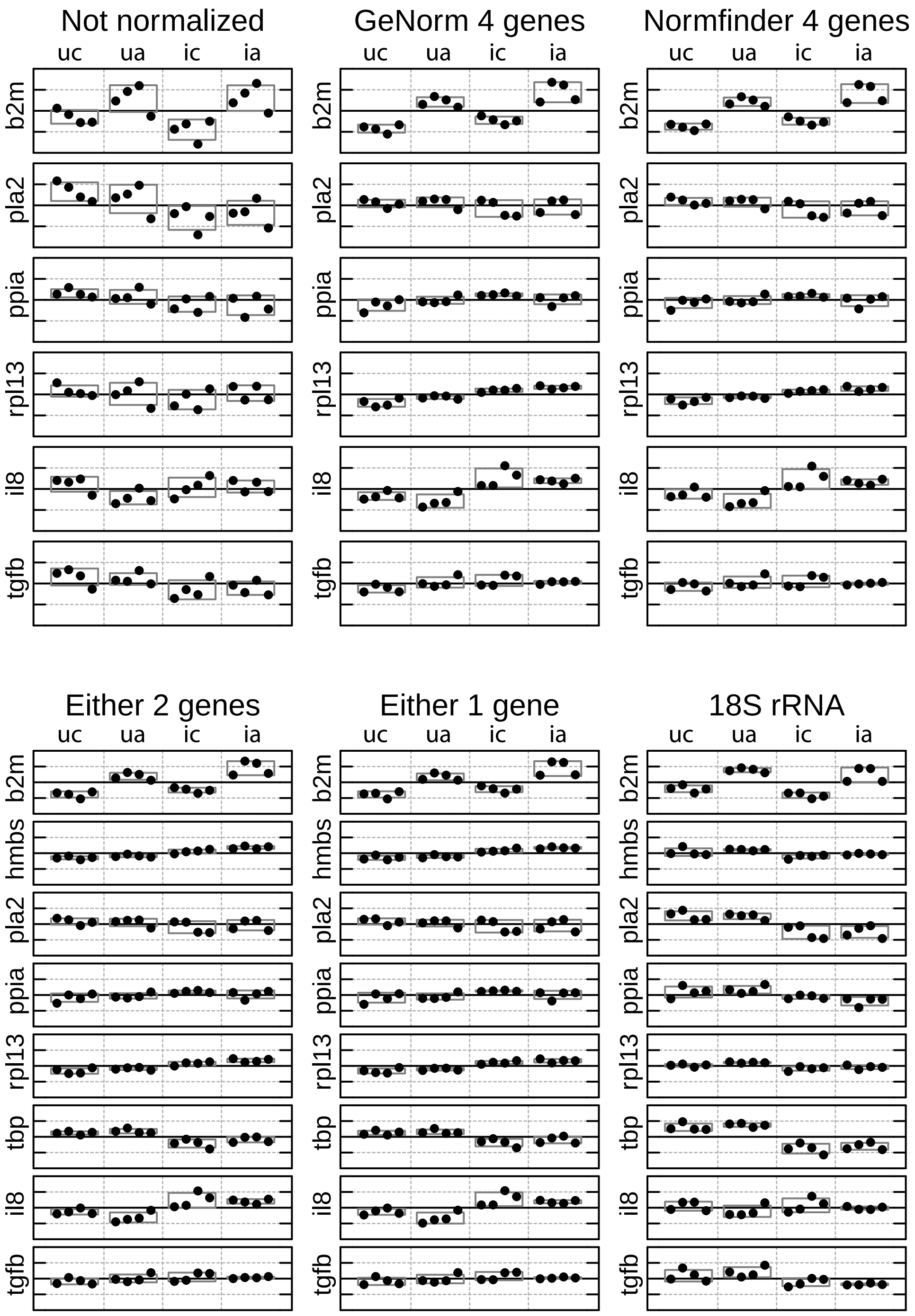

S2 Fig. Residual plots of flu infection experiment data. Four biological replicates of embryo fibroblast were preincubated with (a) or without (c) IFNα, then infected with influenza virus (i) or mock infected (u). Levels of the ten candidate reference transcripts, of 28S rRNA, and of of IL-8 and TGFβ transcripts, were measured by qPCR. Solid circles represent individual sample measure-ments of the transcript indicated to the left, after transformation to a log2 scale (Ct/slope and normalisation, relative to the mean that transcript in all samples, for the six transcripts not used in any normalisation. The vertical axes are marked at unit log2 interval, representing two-fold differ-ences. Different sets of reference transcripts used for normalisation are indicated above each panel of six graphs. Grey boxes show the residual standard deviation of the four replicates from each experimental treatment. All the normalisations show substantial reduction of dispersion of meas-urements within groups, and the removal of sample-specific patterns shared across transcripts. Inclusion of the differentially expressed β2M transcript in the input for the Normfinder reference selection algorithm produced a small but discernable bias when compared with the common refer-ence set selected by GeNorm and by Normfinder after exclusion of β2M. Normalising with rRNA resulted in a clear bias of levels in the infected samples, lowering the relative levels of all tran-scripts compared with those in the uninfected samples.
