# Supplementary material for: A Versatile Panel of Reference Gene Assays for the Measurement of Chicken mRNA by Quantitative PCR
Source: PLoS One. 2016 Aug 18;11(8):e0160173. doi: 10.1371/journal.pone.0160173 (PMC4990416; doi:10.1371/journal.pone.0160173)
Supplement: S3 Fig — (PDF) [file pone.0160173.s003.pdf]

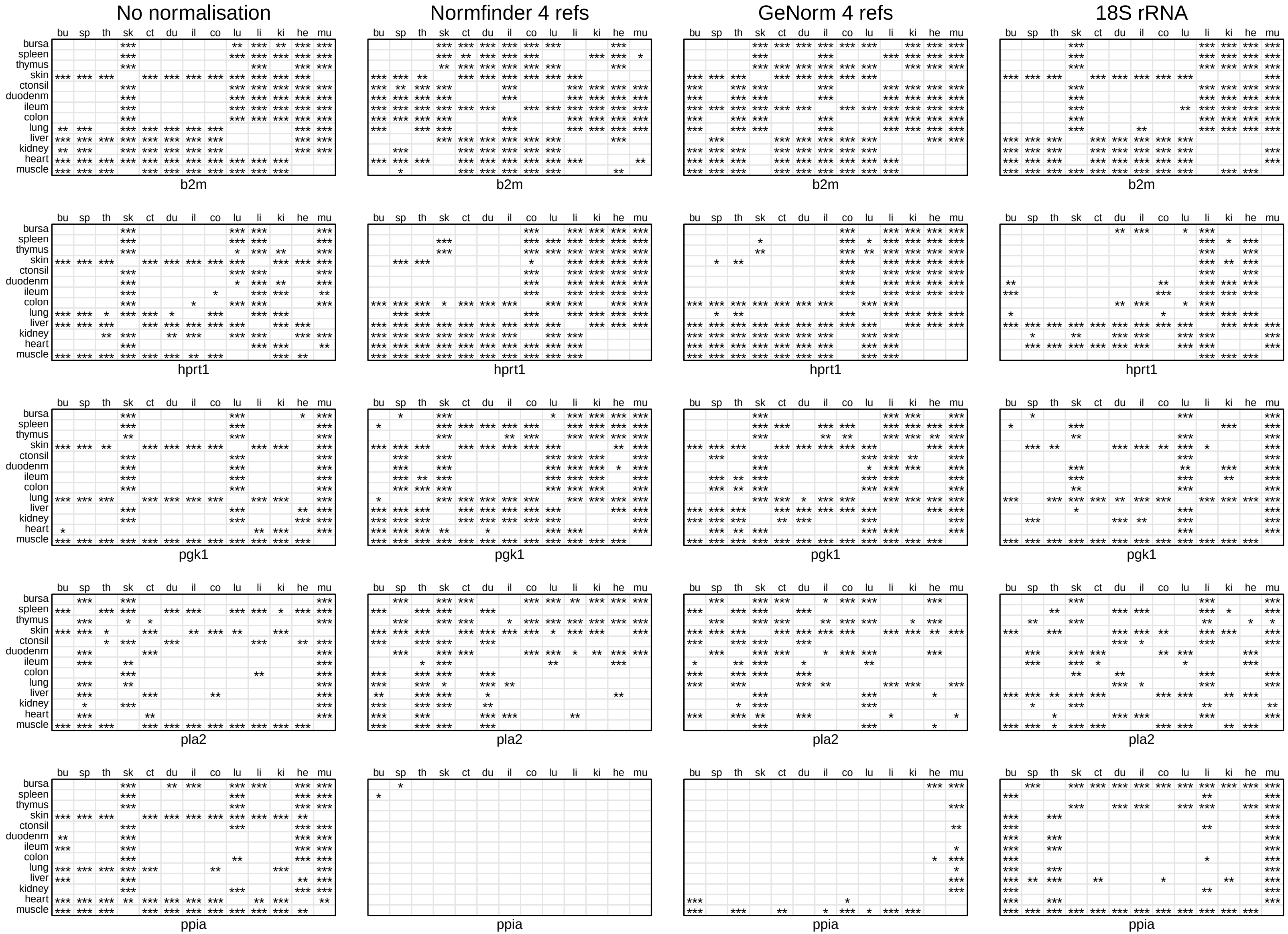

S3 Fig. Effect of normalisation on detection of significant differences in tissue panel. The title above each column indicates the set of genes used as reference genes for normalisation. For Normfinder, the reference genes were ACTB, HMBS, RPLP13 and TBP. For Genorm, ACTB was replaced with RPLP0. Each grid shows the results of statistical analysis for the gene indicat-ed beneath. Asterisks indicate p values for rejection of the null hypothesis that a gene is not differentially expressed in the tissues indicated by row and column labels: *** = p<0.001, ** = p<0.01, * = p<0.05. Column labels are the same as the row labels, but truncated.
