# Supplementary material for: A Versatile Panel of Reference Gene Assays for the Measurement of Chicken mRNA by Quantitative PCR
Source: PLoS One. 2016 Aug 18;11(8):e0160173. doi: 10.1371/journal.pone.0160173 (PMC4990416; doi:10.1371/journal.pone.0160173)
Supplement: S4 Fig — (PDF) [file pone.0160173.s004.pdf]

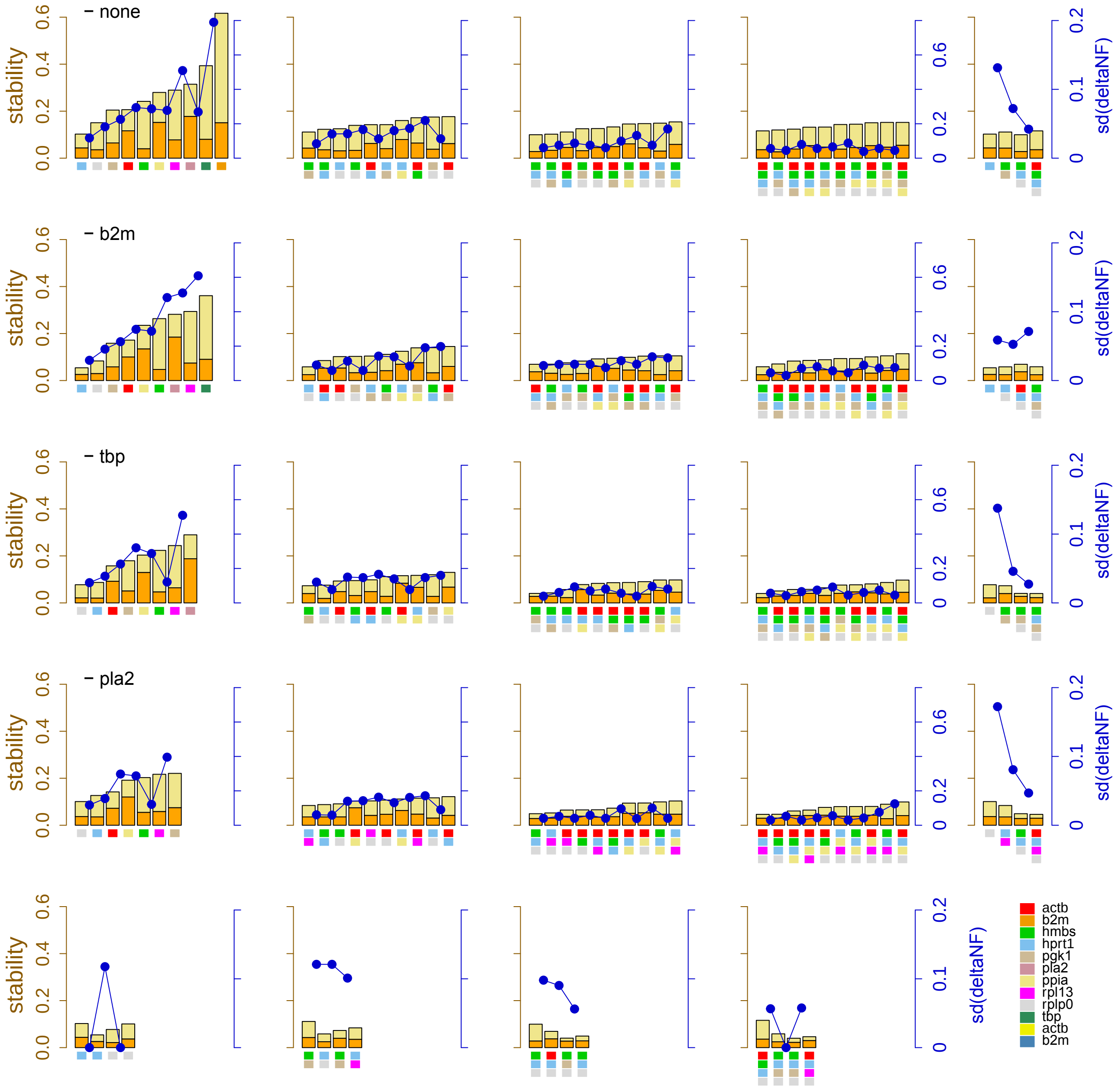

S4 Fig. Nested loop Normfinder reference gene selection (flu infection data). Bar graphs indicate the normfinder rho stability measure for combinations of reference genes in the context of different sets of input data. The intra-group component is orange, and the inter-group component yellow. The genes involved in each combination is shown by the coloured rectangles underneath the bar, which can be decoded in the key at the bottom right. Each successive row of graphs, except the last, presents the results after dropping from the input the least stable (highest rho) gene in the single gene graph in the row above. The last dropped gene is identified above the first graph in each row. Except for the rightmost, sequential graphs in each row present the stability measures for combinations of increasing numbers of genes. Within each graph, combinations are ranked from the most stasble on the left. The graphs on the right compare the stabilities of the most stable combination for increasing numbers of genes. The graphs at the bottom compare the most stable combinations of the same size, for each successively dropped input gene. Blue circles show the standard deviation of the differences in the individual sample normalisation factors between the two combinations they overlap on either side.

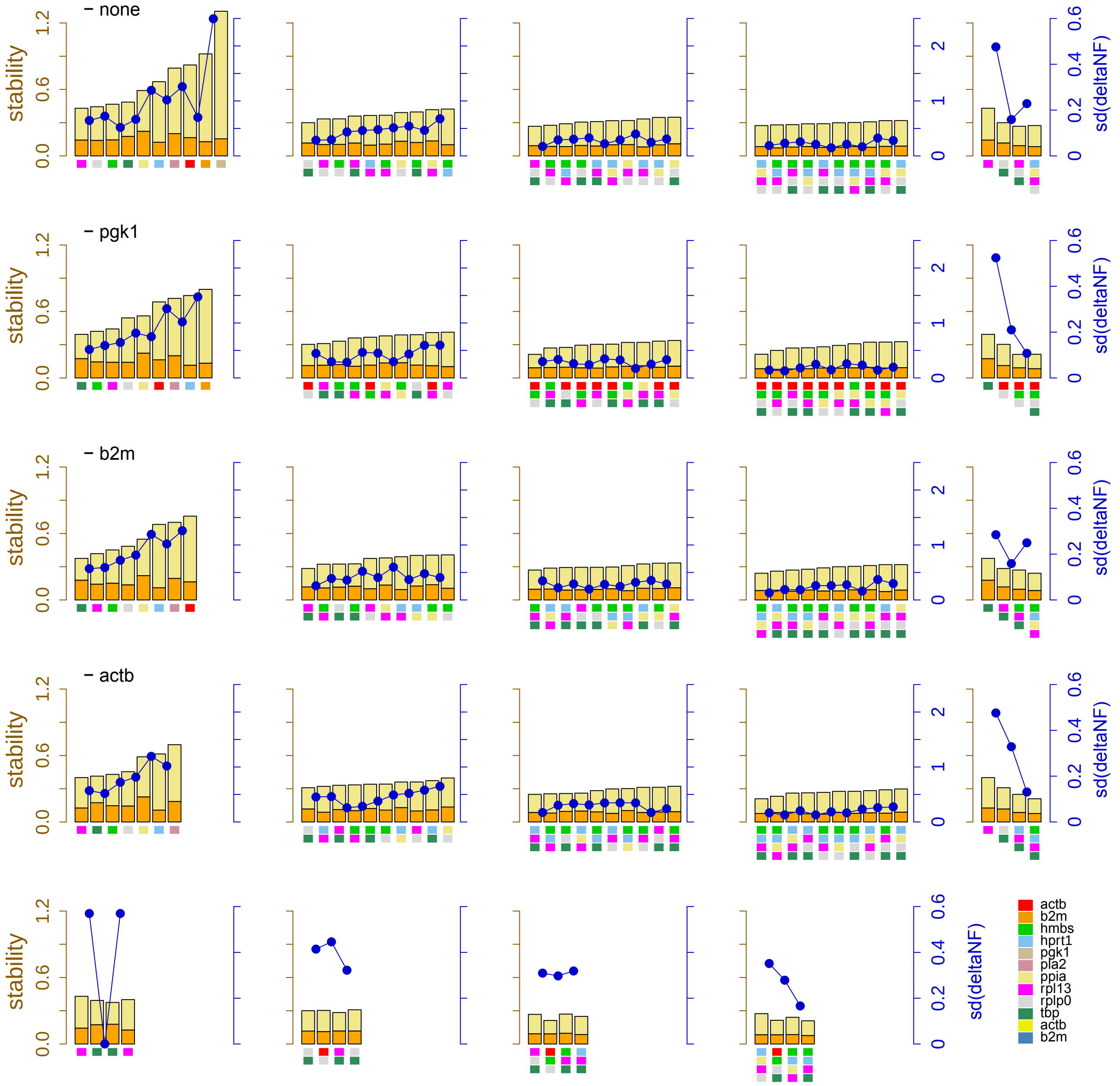

S4 Fig. Nested loop Normfinder reference gene selection (tissue data). Bar graphs indicate the normfinder rho stability measure for combinations of reference genes in the context of different sets of input data. The intra-group component is orange, and the inter-group component yellow. The genes involved in each combination is shown by the coloured rectangles underneath the bar, which can be decoded in the key at the bottom right. Each successive row of graphs, except the last, presents the results after dropping from the input the least stable (highest rho) gene in the single gene graph in the row above. The last dropped gene is identified above the first graph in each row. Except for the rightmost, sequential graphs in each row present the stability measures for combinations of increasing numbers of genes. Within each graph, combinations are ranked from the most stasble on the left. The graphs on the right compare the stabilities of the most stable combination for increasing numbers of genes. The graphs at the bottom compare the most stable combinations of the same size, for each successively dropped input gene. Blue circles show the standard deviation of the differences in the individual sample normalisation factors between the two combinations they overlap on either side.
