# Supplementary figures and images for: A Versatile Panel of Reference Gene Assays for the Measurement of Chicken mRNA by Quantitative PCR
Source: PLoS One. 2016 Aug 18;11(8):e0160173. doi: 10.1371/journal.pone.0160173 (PMC4990416; doi:10.1371/journal.pone.0160173)

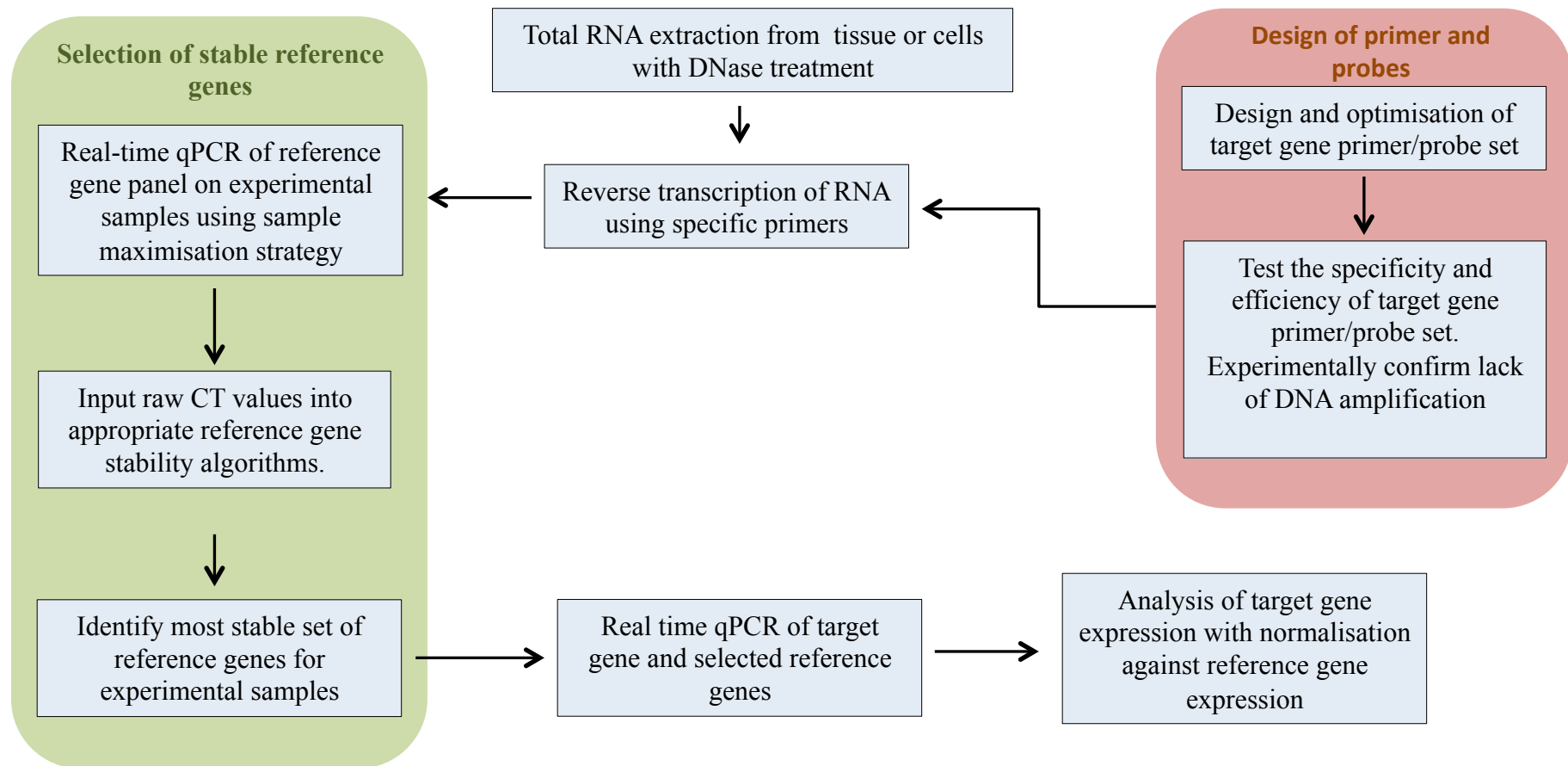

S5 Fig. Reference gene assay design and evaluation flowchart

Supplement: S5 Fig — (PDF) [file pone.0160173.s005.pdf]
