# Supplementary material for: A Versatile Panel of Reference Gene Assays for the Measurement of Chicken mRNA by Quantitative PCR
Source: PLoS One. 2016 Aug 18;11(8):e0160173. doi: 10.1371/journal.pone.0160173 (PMC4990416; doi:10.1371/journal.pone.0160173)
Supplement: S6 Fig — (PDF) [file pone.0160173.s006.pdf]

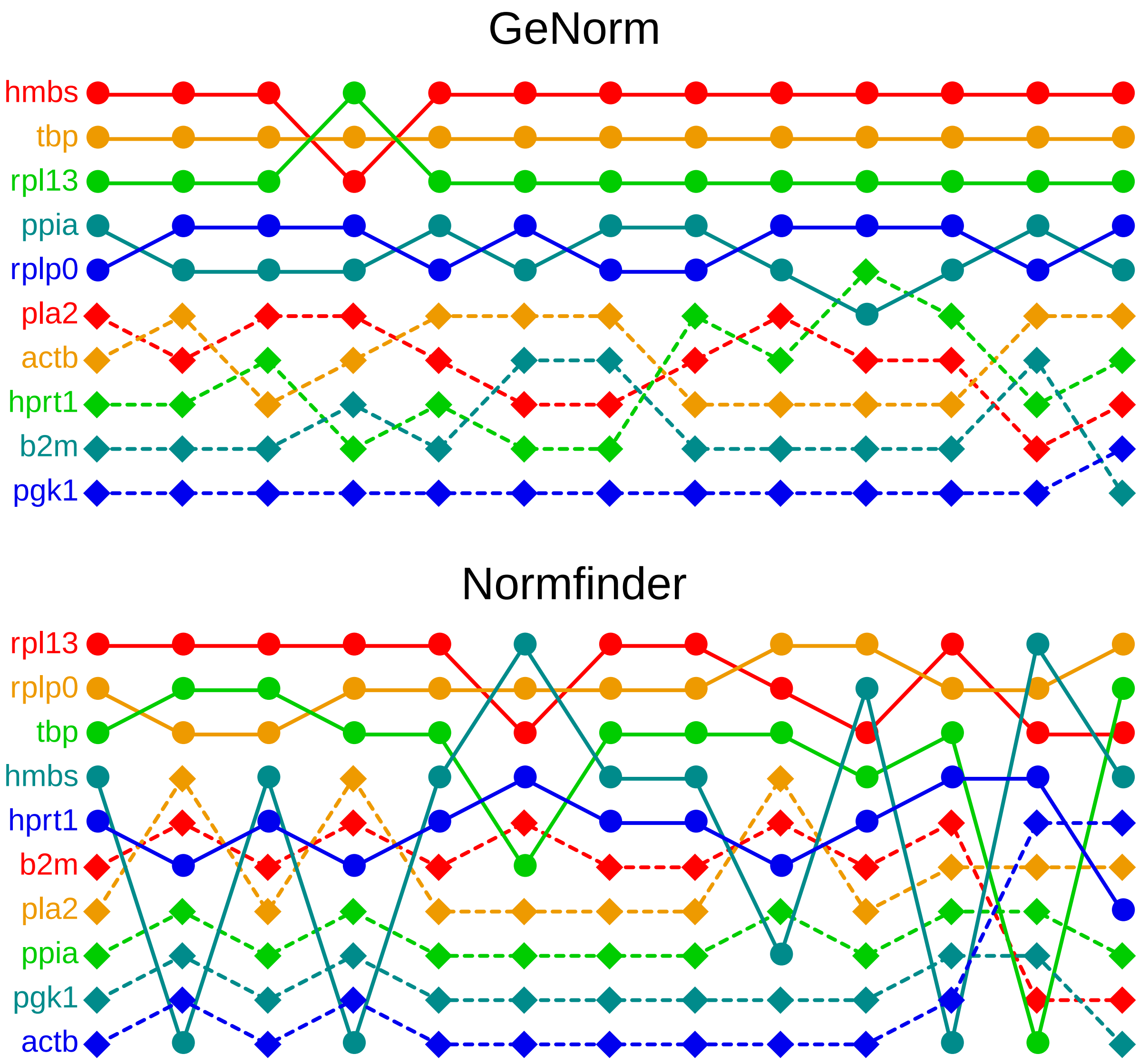

S6 Fig. Robustness of reference gene selection. GeNorm(top) and Normfinder (bottom) stepwise procedures from NormqPCR were applied to the tissue panel dataset after removal of data for one tissue (each removed tissue, left to right). The ranking of each gene in the depleted datasets is shown by the colour and symbol as indicated by the labels to the left of the graphs.
