# Supplementary material for: A Versatile Panel of Reference Gene Assays for the Measurement of Chicken mRNA by Quantitative PCR
Source: PLoS One. 2016 Aug 18;11(8):e0160173. doi: 10.1371/journal.pone.0160173 (PMC4990416; doi:10.1371/journal.pone.0160173)
Supplement: S3 Text — (PDF) [file pone.0160173.s010.pdf]

A. Basic calculations.

The general equation relating Cq C_{Gsn} to amount A of a transcript G in a sample s, assayed on plate n with quantitation thresold level T_{Gn} is …

$$log_2(A_{Gs}) = \frac{C_{Gsn}}{S_{Gn}} + log_2(T_{Gn}) \quad [1]$$

… where S_{An} is the appropriate slope of the regression of $\log_2(A_G)$ vs C. This may be specific for each plate, if a standard curve is included on every plate. The first step in all our calculations is to convert all Cq to the same log_2 scale by dividing by the slope, giving $Q_{Gsn} = C_{Gsn}/S_{Gn}$. In therms of the converted measure equation [1] then becomes …

$$log_2(A_{Gs}) = Q_{Gsn} + log_2(T_{Gn}) \quad [2]$$

We first consider the case where the object of interest is the ratio R of the level of expression in two samples, r and s. Here …

$$log_2(R_{Gr:s}) = log_2\left(\frac{A_{Gr1}}{A_{Gs2}}\right) = Q_{Gr1} - Q_{Gs2} + log_2(T_{G1}) - log_2(T_{G2}) \quad [3]$$

Where the assays are on different plates, it is necessary to include identical control samples for each transcript assayed on each plate, to correct inter-plate differences. Let us call these samples p_i with amounts A_{Gp} and measurements C_{Gp_in} on plate n. Then the geometric mean[1] of the ratios of N_p control sample measurements on the two plates are …

$$\frac{1}{N_p}\sum_{i=1}^{N_p}\left(log_2\left(A_{Gp_in}\right)\right) = \frac{1}{N_p}\sum_{i=1}^{N_p}\left(Q_{Gp_in}\right) + log_2(T_{Gn})$$

$$i.e. \qquad log_2(T_{Gn}) = \frac{1}{N_p}\sum_{i=1}^{N_p}\left(log_2\left(A_{Gp_in}\right)\right) - \frac{1}{N_p}\sum_{i=1}^{N_p}\left(Q_{Gp_in}\right)$$

$$so: \qquad log_2(T_{G1}) - log_2(T_{G2}) = -\frac{1}{N_p}\sum_{i=1}^{N_p}\left(Q_{Gp_i1} - Q_{Gp_i2}\right)$$

$$so\ from\ [3]: \qquad log_2(R_{Gr:s}) = Q_{Gr1} - Q_{Gs2} - \frac{1}{N_p}\sum_{i=1}^{N_p}\left(Q_{Gp_i1} - Q_{Gp_i2}\right) \quad [4]$$

It is worth noting that this inter-plate correction is has exactly the same effect as shifting the threshold. In many cases it may be possible to adjust the threshold in the instrument data export process so that the inter-plate controls yield the same Cqs. If the correcting term is thereby sufficiently reduced, its omission might be justfiable, simplifying further analysis. Of course the best solution, when it is possible, is to carry out all assays for each transcript on the same plate, so that $Q_{Gp_i1} = Q_{Gp_i2}$ and the correction disappears from the equation, which becomes ...

$$log_2(R_{Gr:s}) = Q_{Gr} - Q_{Gs} \quad [5]$$

In this case the inter-plate calibration samples need not be run, although their omission could compromise possible comparisons with later experiments.

So far, the calculation has assumed that the two samples contain the same amount of RNA. This is not generally the case, and samples are usually analysed for amounts of some, presumably constantly expressed, reference gene(s). Then the amounts of the gene of interest are expressed in terms of their ratios to the goemetric mean of the reference genes quantities in the same samples. Now the ratio between two samples becomes* …

$$log_2(R_{Gr:s}) = \left\{Q_{Gr1} - \frac{1}{N_r}\sum_{i=1}^{N_r}\left(Q_{R_irn_i} - log_2\left(T_{R_in_i}\right)\right)\right\} - \left\{Q_{Gs2} - \frac{1}{N_r}\sum_{i=1}^{N_r}\left(Q_{R_isn_i} - log_2\left(T_{R_in_i}\right)\right)\right\} - \frac{1}{N_p}\sum_{i=1}^{N_p}\left(Q_{Gp_i1} - Q_{Gp_i2}\right)$$

$$i.e. \quad log_2(R_{Gr:s}) = (Q_{Gr1} - Q_{Gs2}) - \frac{1}{N_r}\sum_{i=1}^{N_r}\left(Q_{R_irn_i} - Q_{R_isn_i}\right) - \frac{1}{N_p}\sum_{i=1}^{N_p}\left(Q_{Gp_i1} - Q_{Gp_i2}\right) \qquad [6]$$

*note: this equation describes the case where all assays for each reference gene are on the same plate. If not, then further inter-plate controls are required for each, and the equation becomes cumbersome …

$$log_2(R_{Gr:s}) = (Q_{Gr1} - Q_{Gs2}) - \frac{1}{N_p}\sum_{i=1}^{N_p}\left(Q_{Gp_i1} - Q_{Gp_i2}\right) - \frac{1}{N_r}\sum_{i=1}^{N_r}\left\{\left(Q_{R_irn_i} - Q_{R_ism_i}\right) - \frac{1}{N_q}\sum_{j=1}^{N_q}\left(Q_{R_iq_jn_j} - Q_{R_iq_jm_j}\right)\right\} \qquad [7]$$

In the case that all assays for all genes are carried out on the same plates this becomes …

$$log_2(R_{Gr:s}) = (Q_{Gr} - Q_{Gs}) - \frac{1}{N_r}\sum_{i=1}^{N_r}\left(Q_{R_ir} - Q_{R_is}\right) \qquad [8]$$

One important thing to notice about these equations is that all the intercept/threshold terms always cancel out. This means their errors also cancel out. Error-limitation arguments have been advanced in support of carrying out calculations with quantities relative to the mean of experimental Cq (or equivalently, Q). The error in $log_2(A_{As}) = Q_{As} + log_2(T_A)$ is much greater than that in the alternative expression $log_2(A_{As}) = Q_{As} - \overline{Q_{Ax_i}}$, where x_i are all the samples. This is is essentially the same as equation [5], and it describes the ratio of transcript level in sample s to that of an imaginary 'mean' sample. As in the derivation of equation [5], the error in $log_2(T_A)$ cancels out. However, when we go on to calculate the ratios in any two samples, $\overline{Q_{Ax_i}}$ cancels out, just as does $log_2(T_A)$ in the derivation of equation 5. Thus use of the level relative to the mean sample serves no purpose other than to allow the presentation of the intermediate value. The difference between using $\overline{Q_{Ax_i}}$ and $log_2(T_A)$ is essentially a difference in the unit used to express the intermediate values of A_{As}. The choice does not affect the ratios between samples. In both cases, the unit is arbitrary. However, while $log_2(T_A)$ is a

property of the assay, albeit subject to variation, $\overline{Q_{Ax_i}}$ is entirely dependent on the distribution of Cq in the experiment, and even of the subset of samples in any particular analysis of an experiment. This dependence is easily overlooked, which can lead to invalid comparisons, especially between experiments. Therefore we prefer to use $log_2(T_A)$.

These considerations apply equally to the measurement of absolute transcript levels because the only way to arrive at these is by measuring the ratio of a sample to a known standard. Either $\overline{Q_{Ax_i}}$ or $log_2(T_A)$ cancel out in the calculation of that ratio.

A similar compounding of errors happens if the object of our interest is not the comparison of one transcript in different samples, but rather comparison of the ratios between different transcripts in different samples. For example, we might be intersted in the ratio of two cytokine gene transcripts in different animals. The appropriate expression is readily obtained from equation [8] …

$$log_2\left(\frac{R_{(G:H)r}}{R_{(G:H)s}}\right) = log_2\left(\frac{A_{Gr}}{A_{Gs}} \times \frac{A_{Hs}}{A_{Hr}}\right)$$
$$= \left\{(Q_{Gr1} - Q_{Gs2}) - \frac{1}{N_r}\sum_{i=1}^{N_r}\left(Q_{R_irn_i} - Q_{R_isn_i}\right) - \frac{1}{N_p}\sum_{i=1}^{N_p}\left(Q_{Gp_i1} - Q_{Gp_i2}\right)\right\}$$
$$- \left\{(Q_{Hr3} - Q_{Hs4}) - \frac{1}{N_r}\sum_{i=1}^{N_r}\left(Q_{R_irn_i} - Q_{R_isn_i}\right) - \frac{1}{N_p}\sum_{i=1}^{N_p}\left(Q_{Hp_i3} - Q_{Hp_i4}\right)\right\}$$

Now the normalisation terms cancel out, and we get …

$$log_2\left(\frac{R_{(G:H)r}}{R_{(G:H)s}}\right) = (Q_{Gr1} - Q_{Gs2}) - (Q_{Hr3} - Q_{Hs4})$$
$$- \left\{\frac{1}{N_p}\sum_{i=1}^{N_p}\left\{\left(Q_{Gp_i1} - Q_{Gp_i2}\right) - \left(Q_{Hp_i3} - Q_{Hp_i4}\right)\right\}\right\} \qquad [9]$$

If all assays for each gene are on the same plate, the correcting term disappears and we have …

$$log_2\left(\frac{R_{(G:H)r}}{R_{(G:H)s}}\right) = (Q_{Gr} - Q_{Gs}) - (Q_{Hr} - Q_{Hs}) \qquad [10]$$

In either [9] or [10], the normalisation terms have cancelled out. This means that using intermediate normalised values in stepwise error propagation will include the random error in the reference transcript assays which should be cancelled out, uneccesarily inflating the error. So if comparison of ratios of different transcripts in experimental groups is the aim, equation 9 or 10 should be used directly. Normalisation using reference genes is not only uneccesary, but may be deleterious if stepwise error propagation is used.

B. Errors

There are two sources of experimental errors in the calculations described. First is the individual random variability, whether technical or biological, in the individual measurements of Cq. The second is in the slopes of the standard curves that are used to convert Cq into Q. We will discuss the former first.

Random variation arises at multiple levels. For the tissue sample analyses described here we can identify underlying biological variation, sample related variation in the representation of substructures in the tissue architechture, variation in the efficiency of extraction of RNA and variation in the qPCR assay. Where reverse transcription and PCR assays are conducted separately, the latter has variability at two levels, in reverse transcription, shared between different assays on the same sample, and in the PCR reaction, specific to each measurement.

When a normalisation factor is calculated for each sample and does not cancel out in subsequent calculation, that is when comparing levels of a transcript among samples, the error in the reference gene contributes variance to the result of the calculation, so that it is necessary to take account of the random error in the reference transcript assays in any statistical analysis. Hellemans et al. (….) have used error propagation to deal with this requirement. They combine the standard error of the normalisation factor, from replicate assays on each sample, with that from the assays of the gene(s) of interest, using error propagation calculation. Since the normalisation factor is necessarily a propertry of an individual sample, this approach can only deal with the technical variation between replicate assays on the same sample. It cannot include biologiocal variation, variation in sample composition.

In many experimental situations, the variance due to biological variation and/or sampling variation is found to be much greater than the technical variance between assays on a single sample. In such cases, and even when the variances are comparable, if resources limit the number of assays, means may be more accurately measured by maximising biological replicates without nested technical replication of the assays. In that case, the overall error combines the two sources of variance. In the same way, a single reference gene measurement for each sample simply contributes to the overall variance. In experiments of this design, without technical replication, the observed variance represents the sum of biological and technical error in both reference and genes of interest. It is therefore only appropriate in estimating means, not in estimation biological variance per se. If the latter is the objective, technical variance must be measured so that its contribution can be removed. I that case, the estimation of variance by appropriate formulation of the statistical model can be use as an alternative to error propagation methods.

In the work described here, we have maximised biological replication at the expense of technical replication, and the combined errors have been estimated by the statistical modelling rather than by explicit error propagation. This has the further advantage of allowing selection of the most appropriate model for variance using established methods of model comparison.

Effect of errors in standard curve slopes

From equation 7 …

$$log_2(R_{Gr:s}) = (Q_{Gr1} - Q_{Gs2}) - \frac{1}{N_p}\sum_{i=1}^{N_p}\left(Q_{Gp_i1} - Q_{Gp_i2}\right) - \frac{1}{N_r}\sum_{i=1}^{N_r}\left\{\left(Q_{R_irn_i} - Q_{R_ism_i}\right) - \frac{1}{N_q}\sum_{j=1}^{N_q}\left(Q_{R_iq_jn_j} - Q_{R_iq_jm_j}\right)\right\} \qquad [7]$$

$$= \left(\frac{C_{Gr1}}{S_{G1}} - \frac{C_{Gs2}}{S_{G2}}\right) - \frac{1}{N_p}\sum_{i=1}^{N_p}\left(\frac{C_{Gp_i1}}{S_{G1}} - \frac{C_{Gp_i2}}{S_{G2}}\right) - \frac{1}{N_r}\sum_{i=1}^{N_r}\left\{\left(\frac{C_{R_irn_i}}{S_{R_in_i}} - \frac{C_{R_ism_i}}{S_{R_im_i}}\right) - \frac{1}{N_q}\sum_{j=1}^{N_q}\left(\frac{C_{R_iq_jn_j}}{S_{R_in_j}} - \frac{C_{R_iq_jm_j}}{S_{R_im_j}}\right)\right\}$$

$$= \frac{1}{S_{G1}}\left\{C_{Gr1} - \frac{1}{N_p}\sum_{i=1}^{N_p} C_{Gp_i1}\right\} - \frac{1}{S_{G2}}\left\{C_{Gs2} - \frac{1}{N_p}\sum_{i=1}^{N_p} C_{Gp_i2}\right\} - \frac{1}{N_r}\sum_{i=1}^{N_r}\left\{\frac{1}{S_{R_in_i}}\left\{C_{R_irn_i} - \frac{1}{N_q}\sum_{j=1}^{N_q} C_{R_iq_jn_j}\right\} - \frac{1}{S_{R_im_i}}\left\{C_{R_ism_i} - \frac{1}{N_q}\sum_{j=1}^{N_q} C_{R_iq_jm_j}\right\}\right\}$$

The uncertainty in this value, given known uncertainties in values of C and S can be calculated using partial differentials. Calling the value V, and designating uncertainty in quantity X as E_X ...

$$E_V^2 = \left(E_{log_2(R_{Gr:s})}\right)^2$$

$$= \frac{1}{S_{G1}^2}\left\{E_{C_{Gr1}}^2 + \frac{1}{N_p^2}\sum_{i=1}^{N_p} E_{C_{Gp_i1}}^2\right\} + \frac{1}{S_{G2}^2}\left\{E_{C_{Gs2}}^2 + \frac{1}{N_p^2}\sum_{i=1}^{N_p} E_{C_{Gp_i2}}^2\right\}$$

$$+ \frac{1}{N_r^2}\sum_{i=1}^{N_r}\left\{\frac{1}{S_{R_in_i}^2}\left\{E_{C_{R_irn_i}}^2 + \frac{1}{N_q^2}\sum_{j=1}^{N_q} E_{C_{R_iq_jn_j}}^2\right\} + \frac{1}{S_{R_im_i}^2}\left\{E_{C_{R_ism_i}}^2 + \frac{1}{N_q^2}\sum_{j=1}^{N_q} E_{C_{R_iq_jm_j}}^2\right\}\right\}$$

$$+ \frac{E_{S_{G1}}^2}{S_{G1}^4}\left\{C_{Gr1} - \frac{1}{N_p}\sum_{i=1}^{N_p} C_{Gp_i1}\right\}^2 + \frac{E_{S_{G2}}^2}{S_{G2}^4}\left\{C_{Gs2} - \frac{1}{N_p}\sum_{i=1}^{N_p} C_{Gp_i2}\right\}^2$$

$$+ \frac{1}{N_r^2}\sum_{i=1}^{N_r}\left\{\frac{E_{S_{R_in_i}}^2}{S_{R_in_i}^4}\left\{C_{R_irn_i} - \frac{1}{N_q}\sum_{j=1}^{N_q} C_{R_iq_jn_j}\right\}^2 + \frac{E_{S_{R_im_i}}^2}{S_{R_im_i}^4}\left\{C_{R_ism_i} - \frac{1}{N_q}\sum_{j=1}^{N_q} C_{R_iq_jm_j}\right\}^2\right\}$$

In our calculation pipeline so far, slopes have been treated as exactly known constants. In other words, the slope error terms E_{S_x} are all zero. Thus the errors accounted for in the statistical model are those in the first two lines. With constant slopes, $E_{C_{X_{asn}}} = S_{X_{an}} E_{Q_{X_{asn}}}$, so this component is …

$$E_\alpha^2 = E_{Q_{Gr1}}^2 + E_{Q_{Gs2}}^2 + \frac{1}{N_p^2}\sum_{i=1}^{N_p} E_{Q_{Gp_i1}}^2 + \frac{1}{N_p^2}\sum_{i=1}^{N_p} E_{Q_{Gp_i2}}^2 + \frac{1}{N_r^2}\sum_{i=1}^{N_r}\left\{E_{Q_{R_irn_i}}^2 + E_{Q_{R_ism_i}}^2 + \frac{1}{N_q^2}\sum_{j=1}^{N_q} E_{Q_{R_iq_jn_j}}^2 + \frac{1}{N_q^2}\sum_{j=1}^{N_q} E_{Q_{R_iq_jm_j}}^2\right\}$$

This is the error that will be estimated in the statistical model. The remaining error depends only on the error of the slopes. Substituting $C_{X_{sn}}/S_{X_n} = Q_{X_n}$ …

$$E_\beta^2 = \frac{E_{S_{G1}}^2}{S_{G1}^2}\left\{Q_{Gr1} - \frac{1}{N_p}\sum_{i=1}^{N_p} Q_{Gp_i1}\right\}^2 + \frac{E_{S_{G2}}^2}{S_{G2}^2}\left\{Q_{Gs2} - \frac{1}{N_p}\sum_{i=1}^{N_p} Q_{Gp_i2}\right\}^2 + \frac{1}{N_r^2}\sum_{i=1}^{N_r}\left\{\frac{E_{S_{R_in_i}}^2}{S_{R_in_i}^2}\left\{Q_{R_irn_i} - \frac{1}{N_q}\sum_{j=1}^{N_q} Q_{R_iq_jn_j}\right\}^2 + \frac{E_{S_{R_im_i}}^2}{S_{R_in_i}^2}\left\{Q_{R_ism_i} - \frac{1}{N_q}\sum_{j=1}^{N_q} Q_{R_iq_jm_j}\right\}^2\right\} \quad [11]$$

Now $E_V^2 = E_\alpha^2 + E_\beta^2$, where E_α^2 depends only on the errors in Cq and E_β^2 depends only on the proportional errors in the standard curve slopes (as well as differences of mean Q terms).

This is the most complex case. We can simplify in two stages. First by using the same slope for all assays with a given gene, although they may be on more than one plate; then by considering the ideal situation when all assays for a given gene are on the same plate. In the former case, equation [7] remains the same, but in its expansion the coefficients are simplified to …

$$\frac{1}{S_G}\left\{(C_{Gr1} - C_{Gs2}) - \left(\frac{1}{N_p}\sum_{i=1}^{N_p} C_{Gp_i1} - \frac{1}{N_p}\sum_{i=1}^{N_p} C_{Gp_i2}\right)\right\} - \frac{1}{N_r}\sum_{i=1}^{N_r}\frac{1}{S_{R_i}}\left\{(C_{R_irn_i} - C_{R_ism_i}) - \left(\frac{1}{N_q}\sum_{j=1}^{N_q} C_{R_iq_jn_j} - \frac{1}{N_q}\sum_{j=1}^{N_q} C_{R_iq_jm_j}\right)\right\}$$

We will ignore E_α^2, which will be calculated by the statistical model, and deal with E_β^2 …

$$E_\beta^2 = \frac{E_{S_G}^2}{S_G^2}\left\{(Q_{Gr1} - Q_{Gs2}) - \left(\frac{1}{N_p}\sum_{i=1}^{N_p} Q_{Gp_i1} - \frac{1}{N_p}\sum_{i=1}^{N_p} Q_{Gp_i2}\right)\right\}^2 + \frac{1}{N_r^2}\sum_{i=1}^{N_r}\frac{E_{S_{R_i}}^2}{S_{R_i}^2}\left\{(Q_{R_irn_i} - Q_{R_ism_i}) - \left(\frac{1}{N_q}\sum_{j=1}^{N_q} Q_{R_iq_jn_j} - \frac{1}{N_q}\sum_{j=1}^{N_q} Q_{R_iq_jm_j}\right)\right\}^2 \quad [12]$$

With all assays for any transcript on the same plate, we use equation [8] …

$$log_2(R_{Gr:s}) = (Q_{Gr} - Q_{Gs}) - \frac{1}{N_r}\sum_{i=1}^{N_r}(Q_{R_ir} - Q_{R_is}) \quad [8]$$

$$= \frac{1}{S_G}(C_{Gr} - C_{Gs}) - \frac{1}{N_r}\sum_{i=1}^{N_r}\frac{1}{S_{R_i}}(C_{R_ir} - C_{R_is})$$

$$now \quad E_\beta^2 = \frac{E_{S_G}^2}{S_G^4}(C_{Gr} - C_{Gs})^2 + \frac{1}{N_r^2}\sum_{i=1}^{N_r}\frac{E_{S_{R_i}}^2}{S_{R_i}^4}\left(C_{R_i r} - C_{R_i s}\right)^2$$

$$= \frac{E_{S_G}^2}{S_G^2}(Q_{Gr} - Q_{Gs})^2 + \frac{1}{N_r^2}\sum_{i=1}^{N_r}\frac{E_{S_{R_i}}^2}{S_{R_i}^2}\left(Q_{R_i r} - Q_{R_i s}\right)^2 \qquad [13]$$

These equations allow us to discuss the sizes of the part of the error that depends on the slope, and the effects of omitting error from this source on the results obtained from statistical models (which already include the effects of errors in Q). The extent to which the overall error comes from the error in the slopes is dependent on the size of this error compared with the statistically estimated error from biological and technical variability, since the overall error is $\sqrt{E_\alpha^2 + E_\beta^2}$, where…

$$E_\alpha^2 = E_{Q_{Gr}}^2 + E_{Q_{Gs}}^2 + \frac{1}{N_r^2}\sum_{i=1}^{N_r}\left(E_{Q_{R_i r}}^2 + E_{Q_{R_i s}}^2\right) \qquad [14]$$

If all the assays have equal errors, The C_q-associated error E_α will reduce slightly as more reference genes are included, but the effect is small, only about 20% reduction with four reference transcripts. So, as pointed out by Nordgard et al (2006), reduction of variance alone is not sufficient argument for using more than one reference. Rather, the improvement has to depend on the expected reduction of differences in expression between experimental groups, implicitly with GeNorm or explicitly with Normfinder. It remains important that the random error in the reference genes is as small as possible, so that it does not increase the error unecessarily. Nordgard et al. include terms for covariance between assays in their error propagation equations, and showed that in some circumstances, this is necessary for accurate accounting for errors. However, in the cases used here, with each gene assayed only once per biological sample, and with all assays for any gene conducted in a single experiment, we have omitted covariance terms because we consider none will be significantly different from zero.

From equation 13, the overall contribution is essentially two terms, each of which multiplies the squares of a proportional slope error by the squared difference between (adjusted) Cq values. The typical standard error of the slopes for the assays reported here is about 0.02, which corresponds to approximately 1.4% increase or decrease in PCR efficiency. In the two experimental systems described here the lowest standard errors for differences between tissues or experimental groups, calculated by statistical modelling, E_α, are about 0.25, corresponding to about 0.8 to 1.2 fold modulation. These numbers give $E = \sqrt{(0.25)^2 + (0.02 \times \Delta Q)^2}$. With the exception of PGK1 in muscle, the highest absolute ΔQs are about 3, giving $E = \sqrt{(0.25)^2 + (0.02 \times 3)^2} = 0.257$. Even for PGK1 in muscle, inclusion of the slope-related error the error in ΔQ increases it from 0.33 to 0.35. That is from fold differences of about 50.9-80.4 to about 50.2-81.6. Clearly the error component due to the uncertainty of slopes is marginal for this dataset and can safely be ignored when testing for significant differences in transcript levels.

More generally, the slope errors will only be important if either the standard curves are poor or the relevant differences in Cq, for experimental or reference transcripts, are much larger, and the biological and assay variances are small. The equations given can be used to adjust standard errors when that is the case. As a general principle, carrying out assays with similar Cq for reference genes in all samples will mean that slope errors will only be significant when differences in expression of genes of interest are very large. While thay may affect the exact ratios of means for genes of interest,

they are unlikely to affect tests for significance of such large differences. On the other hand, if reference gene measurements are very different, then slope errors may become important.

Looking at the more complex cases, if we have to use inter-plate controls, we should use equation [12]. The change here is the addition of the differences in plate control ΔQ into the terms for each slope error. These should be relatively small. If they are not, the assays must be suspect anyway. They can be minimised by appropriate adjustment of the threshold in the software that provides the Cq. So the discussion of the slope related error in this case is essentially unaltered.

Slopes should not vary between plates. If they do, we have to use equation [11] for the ratios of assays run on different plates. This is very different from [12] and [13], because now it is not ΔQ for a sample that matters, but rather the difference between each value of Q and the plate control(s) of the same transcript. The only way to reduce this error is to have enough plate controls at different concentrations to ensure there is one close to every sample. Of course, to get the plate-specific control, we need to satisfy that requirement anyway, for the standard curve. If these are used for both measuring the slope and inter-plate controls, the standard curve error and the error in the plate controls are no longer independent, and the equation becomes invalid (covariance terms need to be added). Rather than delve into that situation, we would suggest that variation of the standard curve slope on different plates reveals serious problems with the assay. A (possibly partial) standard curve on each plate should still be used to check that the slope is consistent between plates, while it also provides the inter-plate controls. Provided the distribution of indivudual plate slopes care consistent, the curves can be combined to provide a combined curve with lower standard error. Plates with outlier slopes should be discarded.
